# Immunotherapy or targeted therapy as the first-line strategies for unresectable hepatocellular carcinoma: A network meta-analysis and cost-effectiveness analysis

**DOI:** 10.3389/fimmu.2022.1103055

**Published:** 2023-01-11

**Authors:** Kun Liu, Youwen Zhu, Hong Zhu

**Affiliations:** ^1^ Department of Oncology, Xiangya Hospital, Central South University, Changsha, Hunan, China; ^2^ National Clinical Research Center for Geriatric Disorders, Xiangya Hospital, Central South University, Changsha, Hunan, China

**Keywords:** unresectable hepatocellular carcinoma, immunotherapy, targeted therapy, network meta-analysis, cost-effectiveness analysis

## Abstract

**Introduction:**

The existence of many phase III randomized controlled trials (RCTs) of first-line treatment for unresectable hepatocellular carcinoma (HCC) puzzle doctors and patients in choosing the most effective treatment strategies. We aimed to assess the efficacy, safety, and cost-effectiveness of immunotherapy or targeted therapy as the first-line strategy for unresectable HCC.

**Methods:**

The included clinical trials were retrieved from PubMed, Embase, the Cochrane library, and Web of Science databases, in which immunotherapy or targeted therapy was regarded as the first-line treatment for unresectable HCC, published in English between January 1, 2010, and September 20, 2022. We conducted a network meta-analysis (NMA) and cost-effectiveness analysis (CEA) from the Chinese payer’s perspective. Overall survival (OS), progression-free survival (PFS), the ranks of different treatments using P-score, and adverse events (AEs) were evaluated by NMA. Total costs, life-years (LYs), quality-adjusted life-years (QALYs), and incremental cost-benefit ratio (ICER) were estimated from 15-year Markov models developed by CEA.

**Results:**

We identified 2,825 records, including 11,796 patients, from 15 RCTs. The NMA revealed that sintilimab plus a bevacizumab biosimilar (HR, 0.57; 95% CI, 0.43 to 0.75; P = 0.96) and camrelizumab plus rivoceranib (HR, 0.56; 95% CI, 0.41 to 0.66; P = 0.94) could lead to great improvements in OS and PFS compared with sorafenib-related survival. The CEA indicated that tislelizumab increased by 0.220 QALYs (0.312 LYs) and decreased by $1,938 compared with sorafenib, which yielded ICERs of -$8,809/QALY (-$2,612/LY). Sensitivity analysis showed that the model was stable.

**Conclusion:**

Sintilimab plus a bevacizumab biosimilar and camrelizumab plus rivoceranib significantly prolonged OS and PFS, respectively. Further considering the pharmacoeconomics factors, tislelizumab is the most cost-effective first-line treatment strategy for unresectable HCC in China.

## 1 Introduction

Primary liver cancer, including hepatocellular carcinoma (HCC), intrahepatic cholangiocarcinoma and other rare types, was the sixth most commonly diagnosed cancer and the third leading cause of cancer death globally in 2020. Approximately 906,000 new cases of liver cancer were reported globally, out of which 830,000 had a fatal outcome (1). Based on previous data, it is estimated that primary liver cancer will be the fourth most commonly diagnosed cancer and the third leading cause of cancer death in 2022 in China, with 431,383 new cases and 302,327 deaths (2). Although there is a wide variety of treatment methods for primary liver cancer, their efficacy is still unsatisfactory due to the difficulty in early diagnosis, as most patients are diagnosed at the advanced stage of the illness. Currently, some therapeutic approaches include surgery, transarterial chemoembolization, hepatic arterial infusion chemotherapy, radiotherapy, targeted therapy, and immunotherapy (3). However, their efficacy of them is not so desirable. Therefore, it is necessary to recommend the most effective treatment for clinicians and patients to choose.

For patients struggling with unresectable HCC, cancer treatment strategies like targeted therapy and immunotherapy are proven to be effective. In the past decade, numerous immunotherapy drugs and targeted therapy drugs have been gradually tried to apply to the first-line treatment of unresectable HCC, such as brivanib, sunitinib, linifanib, sorafenib, sorafenib plus doxorubicin, sorafenib plus erlotinib, lenvatinib, nivolumab, donafenib, and atezolizumab plus bevacizumab (4–12). Currently, the first-line recommended drugs include sorafenib, lenvatinib, atezolizumab plus bevacizumab, durvalumab, and nivolumab (13). Some regimens have been reported in clinical trials for first-line treatment, like sintilimab plus a bevacizumab biosimilar, cabozantinib plus atezolizumab, tislelizumab, and camrelizumab, durvalumab, pembrolizumab plus lenvatinib (14–19). Additionally, some researchers conducted a comparison of the efficacy of several of them. Liu W et al. found that sintilimab plus a bevacizumab biosimilar was the most effective treatment (20). In addition, another paper demonstrated that atezolizumab plus bevacizumab and sintilimab plus a bevacizumab biosimilar were comparable in efficacy (21). These studies have also become data references for clinical medication.

Following the European Society of Medical Oncology (ESMO) congress, the latest progress of first-line treatment regimens for HCC was updated in 2022, some of which have yielded breakthrough achievements. In the RATIONALE-301 trial, tislelizumab, compared with sorafenib, showed a non-inferiority efficacy in prolonging median overall survival (mOS) [15.9 vs 13.1 months; Hazard ratio (HR), 0.85; 95% confidence interval (CI), 0.712 to 1.019; P = 0.0398] (16). In the SHR-1210-III-310 trial, camrelizumab plus rivoceranib significantly prolonged mOS (22.1 vs 15.2 months; R, 0.62; 95% CI, 0.49 to 0.80; P < 0.0001) and mPFS (5.6 vs 3.7 months; HR, 0.52; 95% CI, 0.41 to 0.65; P < 0.0001) compared with sorafenib (17). In addition, lenvatinib plus pembrolizumab prolonged mOS (21.2 vs 19.0 months; HR, 0.840; 95% CI, 0.708 to 0.997; P = 0.0227) and mPFS (8.2 vs 8.1 months; HR, 0.834; 95% CI, 0.712 to 0.978) compared with lenvatinib alone but did not reach the significance threshold in the LEAP-002 study (19).

Considering that the previous research did not include these regimens comprehensively to compare efficacy indirectly, we conducted this network meta-analysis. However, due to the high morbidity and mortality of primary liver cancer in the Chinese population and most patients diagnosed as an advanced stage at the initial visit, the country and society are facing a huge burden of medical and health care. What’s more, a growing number of novel drugs with expensive cost showed satisfactory efficacy. Therefore, we also performed a cost-effectiveness analysis to evaluate which regimen can balance clinical benefits and medical cost better from Chinese payers’ perspectives.

## 2 Methods

### 2.1 Network meta-analysis

We performed this work according to the PRISMA statement, including a PRISMA NMA checklist (**Supplementary Table 1**).

#### 2.1.1 Study selection and assessment of bias risks

We searched PubMed, Embase, the Cochrane library, and Web of Science for English-language publications from January 1, 2010, to September 20, 2022, with the search terms “nivolumab”, “pembrolizumab”, “atezolizumab”, “camrelizumab”, “durvalumab”, “tislelizumab”, “PD-1”, “PD-L1”, “immunotherapy”, “sorafenib”, “sunitinib”, “linifanib”, “lenvatinib”, “donafenib”, “bevacizumab”, “targeted therapy”, “molecular targeted therapy”, “unresectable hepatocellular carcinoma”, and “clinical trial” (**Supplementary Table 2**). We also retrieved abstracts from the conferences of the European Society of Medical Oncology (ESMO) and the American Society of Clinical Oncology (ASCO). The chosen literature for the conduction of this study should abide by the following inclusion criteria: (1) phase III RCTs for unresectable hepatocellular carcinoma; (2) included immunotherapy or targeted therapy treatment arm instead of locoregional therapy as the first-line treatment; (3) the endpoint included OS and PFS. The exclusion criteria are as follows: (1) a number of patients in the experimental group <10; (2) detailed reports of adverse effects were not available; (3) not published in English. Two independent reviewers (K.L. and Y.W.Z.) screened these studies to exclude repeated articles and those articles not meet the inclusion criteria and extracted relevant data. In the case of disagreement between the two reviewers, we invited a third independent reviewer (H.Z.) for evaluation. We selected the most updated report when there are several reports from the same clinical trial. When we find potentially included abstracts, we first determine whether they meet the inclusion criteria based on their content. In addition, we will contact researchers or the marketing department of the corresponding medical company to obtain detailed data to make the final decision. The bias risk assessment for these clinical trials was performed according to the Cochrane Collaboration guideline (22).

#### 2.1.2 Statistical analysis

The HRs and its 95% CI for OS and PFS of various treatment schemes were obtained using the R software (version 4.1.1, available: http://www.rproject.org) along with the package of “netmeta”. Since there is little data to evaluate the heterogeneity between clinical trials, we established a fixed-effect model. In addition, we compared indirectly the safety of different regimens and calculated the odds ratio (OR) and its 95% CI of all grade adverse events (AEs) and ≥ 3-grade AEs. Finally, we used P-score to rank the efficacy and safety of each regimen.

### 2.2 Cost-effectiveness analysis

We performed this work according to the Consolidated Health Economic Evaluation Reporting Standards (CHEERS) checklist (**Supplementary Table 3**), while this analysis did not involve human subjects or animal study.

#### 2.1.1 Patients and treatments

The patients have received the first-line treatment of various regimens. In the case of progressed disease (PD) or intolerable AEs, patients received regorafenib recommended by the National Comprehensive Cancer Network (NCCN^®^) guidelines and the Chinese Society of Clinical Oncology (CSCO) guidelines as subsequent therapy (13, 23, 24). On the other hand, the remaining patients received the best supportive care (BSC) until death, and those who reached death received terminal care. The specific use of different drugs is detailed in **Supplementary Table 4**. According to these RCTs and the published articles, we assumed that patients were 65 kg in weight, and 164 cm in height with a body surface area of 1.72 m^2^ (25, 26) (**Table 1**).

**Table 1:** Model Parameters: Clinical and Cost data.

Parameters	Baseline value	Range		Reference	Distribution
		Minimum	Maximum		
Clinical data					
Weibull survival model of sorafenib					
OS	Scale= 0.05419, Shape= 1.060795	–	–	(27)	–
PFS	Scale= 0.21713, Shape= 0.81887	**-**	**-**	(27)	**-**
**HRs of other regimens versus sorafenib**	See the results of network meta-analysis				–
Proportion (%) of receiving active second-line treatment					
Brivanib	0.210	0.168	0.252	(4)	Beta
Donafenib	0.372	0.298	0.446	(11)	Beta
Durvalumab	0.432	0.346	0.518	(18)	Beta
Lenvatinib	0.330	0.264	0.396	(9)	Beta
Linifanib	0.363	0.290	0.436	(6)	Beta
Nivolumab	0.380	0.304	0.456	(10)	Beta
Sorafenib	0.398	0.318	0.478	(4–12, 14–19)	Beta
Sunitinib	0.766	0.613	0.919	(5)	Beta
Tislelizumab	0.485	0.388	0.582	(16)	Beta
Atezolizumab plus bevacizumab	0.360	0.288	0.432	(12)	Beta
Cabozantinib plus atezolizumab	0.200	0.160	0.240	(15)	Beta
Camrelizumab plus rivoceranib	0.397	0.318	0.476	(17)	Beta
Pembrolizumab plus lenvatinib	0.441	0.353	0.529	(19)	Beta
Sintilimab plus IBI305	0.290	0.232	0.348	(14)	Beta
Sorafenib plus erlotinib	0.450	0.360	0.540	(8)	Beta
Sorafenib plus doxorubicin	0.728	0.582	0.874	(7)	Beta
Risk of main AEs in lenvatinib group					
Risk of decreased appetite	0.046	0.037	0.055	(9)	Beta
Risk of AST increased	0.050	0.040	0.060	(9)	Beta
Risk of thrombocytopenia	0.055	0.044	0.066	(9)	Beta
Risk of proteinuria	0.057	0.046	0.068	(9)	Beta
Risk of decreased weight	0.076	0.061	0.091	(9)	Beta
Risk of hypertension	0.233	0.186	0.280	(9)	Beta
Risk of main AEs in tislelizumab group					
Risk of AST increase	0.059	0.047	0.071	(16)	Beta
**Risk of main AEs in orther group**	See the results of randomized controlled trials			(4–8, 10–12, 14, 15, 17–19)	Beta
Utility					
PFS	0.760	0.608	0.912	(27)	Beta
PD	0.680	0.544	0.60	(27)	Beta
Disutility due to AEs (grade ≧̸3)	0.160	0.128	0.192	(28)	Beta
Cost data (US, $)					
**Drug cost, $/per cycle**					
Brivanib	10,769	8,615	12,923	Local Charge	Gamma
Donafenib	2,204	1,763	2,645	Local Charge	Gamma
Durvalumab	2,625	2,100	3,150	Local Charge	Gamma
Lenvatinib	1,975	1,580	2,370	Local Charge	Gamma
Linifanib	7,601	6,081	9,121	Local Charge	Gamma
Nivolumab	2,515	2,012	3,018	Local Charge	Gamma
Sorafenib	2,422	1,938	2,906	Local Charge	Gamma
Sunitinib	1,312	1,050	1,574	Local Charge	Gamma
Tislelizumab	842	674	1010	Local Charge	Gamma
Atezolizumab plus bevacizumab	6,624	5,299	7,945	Local Charge	Gamma
Cabozantinib plus atezolizumab	6,330	5,064	7,596	Local Charge	Gamma
Camrelizumab plus rivoceranib	1,832	1,466	2,198	Local Charge	Gamma
Pembrolizumab plus lenvatinib	3,275	2,620	3,930	Local Charge	Gamma
Sintilimab plus IBI305	3,819	3,055	4,583	Local Charge	Gamma
Sorafenib plus erlotinib	2,974	2,379	3,569	Local Charge	Gamma
Sorafenib plus doxorubicin	2,490	1,992	2,988	Local Charge	Gamma
Regorafenib	3,154	2,523	3,785	Local Charge	Gamma
Cost of AEs					
Brivanib	74	59	89	(29–32)	Gamma
Donafenib	4	3	5	(29, 31)	Gamma
Durvalumab	5	4	6	(30)	Gamma
Lenvatinib	42	34	50	(29–32)	Gamma
Linifanib	96	77	115	(29–32)	Gamma
Nivolumab	6	5	7	(30)	Gamma
Sorafenib	13	10	16	(29–31)	Gamma
Sunitinib	157	126	188	(29–33)	Gamma
Tislelizumab	6	5	7	(30)	Gamma
Atezolizumab plus bevacizumab	9	7	11	(29, 30)	Gamma
Cabozantinib plus atezolizumab	22	18	26	(29–31)	Gamma
Camrelizumab plus rivoceranib	117	94	140	(29–33)	Gamma
Pembrolizumab plus lenvatinib	26	21	31	(29–31)	Gamma
Sintilimab plus IBI305	49	39	59	(29–32)	Gamma
Sorafenib plus erlotinib	154	123	185	(27, 29, 30, 33)	Gamma
Sorafenib plus doxorubicin	81	65	97	(29, 31–34)	Gamma
**Follow-up and monitoring per cycle**	228	182	274	(35)	Gamma
**Best supportive care per cycle**	423	338	508	(30)	Gamma
**Terminal care per patient**	2,035	1,628	2,442	(35)	Gamma
**Body weight (kilogram)**	65	52	78	(25, 26)	Normal
**Body surface area (meters^2^ )**	1.72	1.38	2.06	(25, 26)	Normal
**Discount rate**	0.03	0	0.05	(34)	Uniform

OS, overall survival; PFS, progression-free survival; IBI305, bevacizumab biosimilar; AEs, adverse events; AST, aspartate aminotransferase; NA, not applicable.

#### 2.2.2 Model structure

To evaluate the cost-effectiveness of the different first-line regimens of patients with unresectable HCC, we constructed a Markov model with a length of 6 weeks and three health states (PFS, progressive disease (PD), and death) (**Supplementary Figure 1**) using TreeAge Pro 2020 (TreeAge Software, Williamstown, MA, https://www.treeage.com) and the the time horizon was 15 years. The costs and effects were discounted at a rate of 3% per year (34). The outputs we measured included the total cost, life-years (LYs), quality-adjusted LYs (QALYs), and incremental cost-effectiveness ratios (ICERs). The willingness-to-pay (WTP) was $37,653 per QALY in China (25).

#### 2.2.3 Utility estimates

We collected data from the OS and PFS curves from included RCTs using GetData Graph Digitizer (version 2.26; http://www.getdata-graph-digitizer.com/index.php), whereas the Weibull distribution was chosen as the best-fitting parameter model. This selection was made among well-known models such as log-logistic, Gompertz, Weibull, exponential, and log-normal distribution according to Akaike’s information criterion (AIC) and Bayesian information criterion (BIC) (**Supplementary Figure 2** and **Table 5**) (36). As for sorafenib, we reconstructed the survival curves according to the survival data reported by each clinical trial. We got two parameters, shape (γ) and scale (λ), using R software (25). We used previously published utilities of 0.76 and 0.68 as the mean health utility value for the PFS and the PD state, respectively (27). We also consider the disutility values of grade 3/4 AEs in our analysis (28).

#### 2.2.4 Cost inputs

Direct medical costs were taken solely into account from the Chinese payers’ perspective, including costs of drugs, AEs management (grade 3 or higher AEs with an incidence rate higher than 5%) (27, 29–34), BSC, terminal care, follow-up, and monitoring (30, 35). The drug price and part of the cost of AEs management are from Xiangya Hospital of Central South University. The remaining costs were derived from previously published literature (**Table 1**). All costs are exchanged into US dollars at the rate of *$1 = ¥6.8917*.

#### 2.2.5 Sensitivity analyses

We performed one-way sensitivity analysis and probabilistic sensitivity analysis to evaluate the uncertainty of the model results. One-way sensitivity analysis was conducted within a variance of 20% from their baseline values according to varied values of a certain parameter. We also performed a probabilistic sensitivity analysis to assess the probability of effectiveness of the treatment regimens through 10000 Monte Carlo repetitions.

## 3 Results

### 3.1 Network meta-analysis

**Figure 1:**
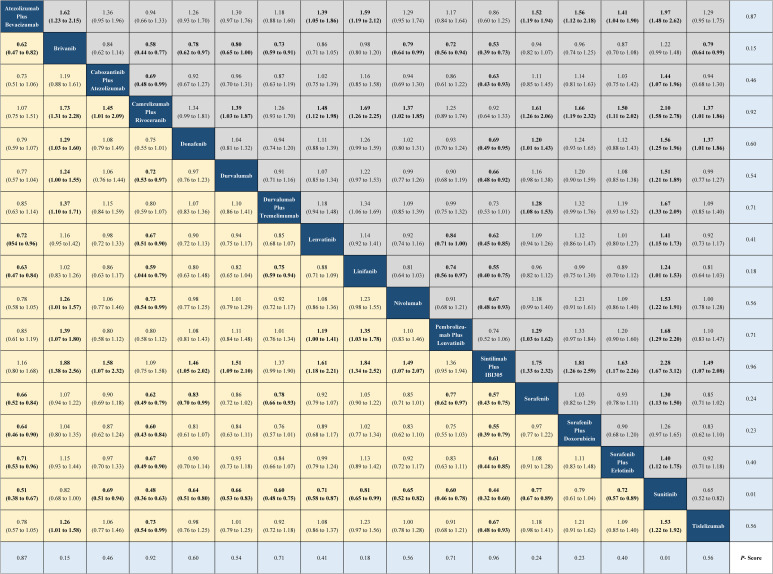
Network meta-analysis for overall survival.

**Figure 2:**
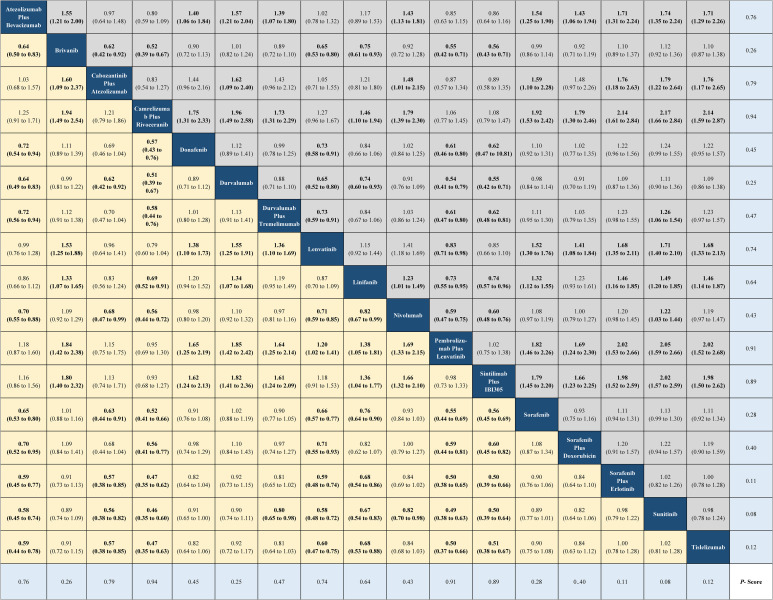
Network meta-analysis for progression-free survival.

The current NMA was conducted upon 15 phase III RCTs in which 2,825 records were screened and 11,796 patients were enrolled (**Supplementary Figure 3**). The model diagram of the NMA is shown in **Supplementary Figure 4**. These trials involved regimens brivanib (N = 577), donafenib (N = 328), durvalumab (N = 389), lenvatinib (N = 877), linifanib (N = 514), nivolumab (N = 371), sunitinib (N = 530), tislelizumab (N = 342), atezolizumab plus bevacizumab (N = 336), cabozantinib plus atezolizumab (N = 432), camrelizumab plus rivoceranib (N = 272), durvalumab plus tremelimumab (N = 393), pembrolizumab plus lenvatinib (N = 395), sintilimab plus a bevacizumab biosimilar (N = 381), sorafenib plus doxorubicin (N = 180), sorafenib plus erlotinib (N = 358), and sorafenib (N = 4,925) (**Supplementary Table 6**). The risk of bias is shown in **Supplementary Table 7**. From the indirect comparisons of the NMA, sintilimab plus a bevacizumab biosimilar (HR, 0.57; 95% CI, 0.43 to 0.75 or 1.75; 1.33 to 2.32; P-score = 0.96) and camrelizumab plus rivoceranib (HR, 0.56; 95% CI, 0.41 to 0.66 or 1.92; 1.53 to 2.42; P-score = 0.94) could lead to great improvements in OS and PFS compared with the sorafenib-related survival (**Figures 1** and **2**). The HRs for OS and PFS of active treatment compared with the sorafenib treatment are shown in **Figures 1** and **2**. The forest plot revealed that tislelizumab had a lower likelihood of all-grade (OR, 0.14; 95% CI, 0.07 to 0.25; P-score = 0.05) and grade 3 or higher AEs (0.25; 0.18 to 0.35; P-score = 0.05) than those of sorafenib, respectively (**Supplementary Figure 5**).

### 3.2 Cost-effectiveness analysis

#### 3.2.1 Base-case analysis

A Markov model was composed of 11,403 participants with a 15-year time horizon, in which the durvalumab plus tremelimumab group was excluded. The total costs and QALYs (LYs) of brivanib, sunitinib, linifanib, sorafenib plus doxorubicin, sorafenib plus erlotinib, lenvatinib, cabozantinib plus atezolizumab, durvalumab, tislelizumab, nivolumab, donafenib, pembrolizumab plus lenvatinib, camrelizumab plus rivoceranib, atezolizumab plus bevacizumab, sintilimab plus a bevacizumab biosimilar, and sorafenib were $64,723 and 0.374 (0.524), $26,922 and 0.945 (1.379), $59,860 and 1.379 (1.681), $38,052 and 1.186 (1.717), $35,787 and 1.297 (1.888), $28,080 and 1.367 (1.942), $56,396 and 1.410 (1.994), $33,972 and 1.498 (2.128), $26,808 and 1.509 (2.149), $32,703 and 1.515 (2.148), $31,063 and 1.535 (2.180), $44,731 and 1.594 (2.260), $40,307 and 1.795 (2.603), $73,457 and 1.870 (2.646), $56,259 and 2.076 (2.950), and $28,746 and 1.289 (1.837), which yielded ICERs of -$39,319 (-$27,401), $5,302 ($3,983), -$250,919 (-$199,449), -$83,838 (-$77,550), $138,059 ($880,125), -$3,680 (-$6,343), $228,512 ($176,115), $25,005 ($17,959), -$8,809 (-$2,612), $17,509 ($12,724), $9,419 ($6,755), $52,410 ($37,790), $22,848 ($15,093), $76,955 ($55,267), and $34,959 ($24,675) per QALY (LY) gained than sorafenib, respectively (**Table 2**). Our results demonstrate that tislelizumab or lenvatinib versus sorafenib as first-line systematic treatment were dominant. Further pairwise comparative analysis, treatment with tislelizumab produced an additional 0.142 QALYs (0.207 LYs) and a cost reduction of $1,272 compared with lenvatinib, resulting in an ICER of -$8,958/QALY (-6,145/LY). A comparison of ICER’s pairwise treatment strategies is shown in **Supplementary Table 8**. In summary, tislelizumab was cost-effective as the first-line strategy for unresectable HCC in China.

**Table 2:** Baseline results.

Strategy	LYs	QALYs	Total cost $	ICER $/LY^a^	ICER $/QALY^a^
Brivanib	0.524	0.374	64,723	Dominated^b^	Dominated
Sunitinib	1.379	0.945	26,922	3,983	5,302
Linifanib	1.681	1.165	59,860	Dominated	Dominated
Sorafenib plus Doxorubicin	1.717	1.186	38,052	Dominated	Dominated
Sorafenib	1.837	1.289	28,746	–	–
Sorafenib plus Erlotinib	1.888	1.297	35,787	138,059	880,125
Lenvatinib	1.942	1.367	28,080	Dominant^c^	Dominant
Cabozantinib plus Atezolizumab	1.994	1.410	56,396	176,115	228,512
Durvalumab	2.128	1.498	33,972	17,959	25,005
Tislelizumab	2.149	1.509	26,808	Dominant	Dominant
Nivolumab	2.148	1.515	32,703	12,724	17,509
Donafenib	2.180	1.535	31,063	6,755	9,419
Pembrolizumab plus Lenvatinib	2.260	1.594	44,731	37,790	52,410
Camrelizumab plus Rivoceranib	2.603	1.795	40,307	15,093	22,848
Atezolizumab plus Bevacizumab	2.646	1.870	73,457	55,267	76,955
Sintilimab plus IBI305	2.950	2.076	56,259	24,675	34,959

^a^ Compared to sorafenib.

^b^ Other strategies showed lower effectiveness and high cost, as compared with the sorafenib (Dominated).

^C^ Other strategies showed higher effectiveness and lower cost, as compared with the sorafenib (Dominant).

ICER, incremental cost-effectiveness ratio; LY, life-year; QALY, quality-adjusted life-year; IBI305, bevacizumab biosimilar.

#### 3.2.2 Sensitivity analysis

**Figure 3:**
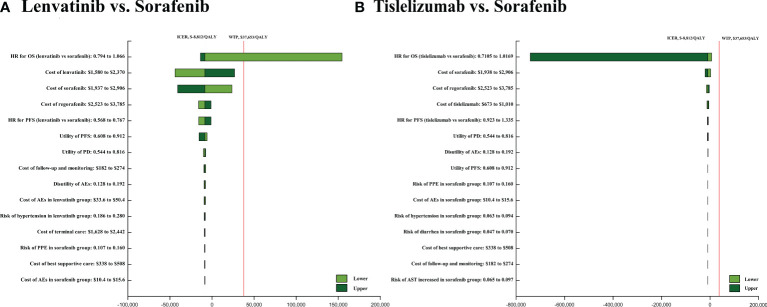
The one-way sensitivity analyses of lenvatinib vs sorafenib **(A)**, tislelizumab vs. sorafenib **(B)**. PFS, progression-free survival; PD, disease progressed; AEs, adverse events.

**Figure 4:**
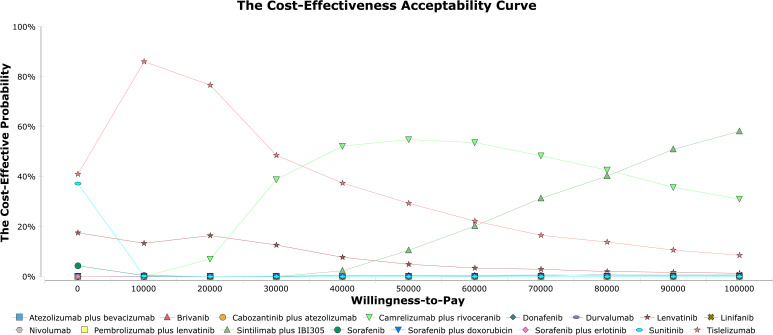
The cost-effectiveness acceptability curves.

The results of one-way sensitivity showed that HRs of OS of the tislelizumab versus sorafenib when comparing tislelizumab and sorafenib, followed by the cost of sorafenib, regorafenib, and tislelizumab. When comparing lenvatinib and sorafenib, HRs of OS of the lenvatinib versus sorafenib, followed by the costs of lenvatinib, sorafenib and regorafenib (**Figure 3**). In the probabilistic sensitivity analysis, the acceptability curve showed that for different WTP values, the most cost-effective solutions are also different (**Figure 4**). For example, tislelizumab is a better choice when it is less than the WTP threshold of $37,653/QALY in China. When the WTP value is between $37,653/QALY and $80,000/QALY, camrelizumab plus rivoceranib is the better choice. When WTP is greater than $80,000/QALY, sintilimab plus a bevacizumab biosimilar is a better choice (**Figure 4**). The scatter plot showed that the probability of tislelizumab, lenvatinib, donafenib, nivolumab, camrelizumab plus rivoceranib, durvalumab, sintilimab plus a bevacizumab biosimilar, pembrolizumab plus lenvatinib, sorafenib plus erlotinib, sunitinib, atezolizumab plus bevacizumab, brivanib, linifanib, sorafenib plus doxorubicin, cabozantinib plus atezolizumab therapies being cost-effective were 96.7%, 86.3%, 84.5%, 82.4%, 75.4%, 73.6%, 60.0%, 24.0%, 0.3%, 1.6%, 0.9%, 0%, 0%, 0%, and 0% compared with sorafenib at a WTP threshold of $37,653/QALY, respectively (**Supplementary Figure 6**).

## 4 Discussion

At present, the morbidity and mortality of primary liver cancer, especially HCC, are very high in the world, and most patients are in the advanced stage. Among all regions of the world, the highest incidence of HCC has been observed in Asia, and China has recorded many cases (37). Furthermore, Chinese patients with unresectable HCC face substantial financial pressure due to medical expenses. From 2012 to 2014, the expenditures of an HCC patient were ¥55,529 ($8,057) in 13 Chinese provinces on average, which included ¥4,592 ($666) of non-medical and the rest ¥50,937 ($7,391) of medical expenditures (38). Due to the poor prognosis and medical care burden of unresectable HCC, numerous clinical trials are conducted to explore an effective treatment option (16, 17, 19, 37). For the moment, there are a large quantity of novel drugs for unresectable HCC to choose, such as brivanib, sunitinib, linifanib, sorafenib plus doxorubicin, sorafenib plus erlotinib, lenvatinib, cabozantinib plus atezolizumab, durvalumab, tislelizumab, nivolumab, donafenib, pembrolizumab plus lenvatinib, camrelizumab plus rivoceranib, atezolizumab plus bevacizumab, sintilimab plus a bevacizumab biosimilar, and sorafenib. Although these therapeutic strategies are effective for treating unresectable HCC, the high cost of these drugs places a heavy burden on social health resources and patients. In addition, there is a lack of head-to-head clinical trials of 16 treatment strategies to show which one is better, and fewer studies to consider its overall cost-effectiveness. Therefore, we performed the first well-rounded network meta-analysis and cost-effectiveness analysis of first-line systemic regiments for unresectable HCC to facilitate treatment strategies for patients and clinicians.

Additionally, there is a lack of head-to-head clinical trials to determine which one was the best choice. Even more, only a few studies discussed the overall cost-effectiveness of each strategy separately. Therefore, this was the first well-rounded NMA and cost-effectiveness analysis evaluating the first-line systematic regimens for unresectable HCC to facilitate treatment strategies for both patients and clinicians.

The last ten years have shown that many novel drugs have been approved to treat irresectable liver cancer. Sorafenib was the first approved molecularly-targeted drug and was regarded as a standard in improving the prognosis of patients struggling with unresectable HCC. In this study, we also uniformly selected sorafenib as a control. However, our findings differed slightly from those of published research (21). Network meta-analysis results revealed that sintilimab plus a bevacizumab biosimilar showed the best effectiveness in prolonging OS, followed by camrelizumab plus rivoceranib and atezolizumab plus bevacizumab. On the other hand, camrelizumab plus rivoceranib ranked the first in prolonging PFS, followed by pembrolizumab plus lenvatinib and sintilimab plus a bevacizumab biosimilar. The anti-angiogenesis effect of immunotherapy combined with targeted therapy is obvious, which highlights the characteristics and advantages of immunotherapy, and gradually changes the existing clinical standard and treatment mode of unresectable HCC. However, all grade and grade 3 or higher AEs in camrelizumab plus rivoceranib group is much higher than other groups. The grade 3 or higher AEs in camrelizumab plus rivoceranib group included hypertension, hepatic insufficiency, palmar-plantar erythro-dysesthesia, and so on, most of which are related to TKI (17). Meanwhile, all grade and grade 3 or higher AEs in the immunotherapy alone group are much lower than other group, such as durvalumab, tislelizumab, and nivolumab. The latter indicated the possibility of immunotherapy being a new trend in curing patients with unresectable HCC. In conclusion, NMA provides strong evidence for the efficacy of immunotherapy in combination with targeted therapy as the first-line treatment for unresectable HCC. Our estimates of survival suggest that the efficacy of immunotherapy plus targeted therapy is better than others. Although immunotherapy remains the most favorable safety, the increased efficacy of combination therapy comes at the cost of a high risk of toxicity, which often results in permanent discontinuation. Therefore, doctors and patients need to consider both efficacy and safety when selecting treatment options according to the patient’s condition in clinical practice.

It is a cost-based innovative treatment strategy that we need to take into consideration in China, an economic powerhouse. The baseline results of the cost-effectiveness analysis indicated that tislelizumab and lenvatinib increased by 0.220 and 0.078 QALYs and decreased by $1,938 and $666 compared with sorafenib, respectively. What’s more, tislelizumab increased by 0.142 QALYs and decreased by $1,272 compared with lenvatinib. The treatment of and sintilimab plus a bevacizumab biosimilar, atezolizumab plus bevacizumab, and camrelizumab plus rivoceranib are related to better efficacy in all first-line strategy, producing 2.076, 1.870, and 1.795 QALYs respectively, which are accordance with the results of NMA. However, considering the medical expenditure of all treatment strategies, tislelizumab is the dominant cost-effective strategies as the first-line treatment for unresectable HCC. The main reasons were: first, considering the affordability of Chinese patients, the price of tislelizumab negotiated with the government is the lowest among all first-line treatment. Second, the costs of dealing with AEs were the least due to the low incidence of grade 3 or higher AEs. Third, patients receiving BSC, who are intolerant to standard treatment strategies for unresectable HCC, accounted for a relatively small percentage.

Our cost-effectiveness analysis is sensitive to the relative efficacy of the first-line treatment for unresectable HCC. The analysis suggested that the economic outcome of lenvatinib became more favorable in patients with lower HR of OS compared with sorafenib and worse in patients with higher HR. However, regardless of whether the HR of OS was higher or lower in tislelizumab group compared with sorafenib, the economic outcome was favorable. This finding is similar to previously published researches, in which the HR of OS is the most influential factor (28, 30, 39–42). Changes in WTP values also affect economic outcomes. Tislelizumab was the most cost-effective treatment option at a WTP threshold of $37,653 per QALY, whereas camrelizumab plus rivoceranib was a preferable option at a WTP threshold of $37,653 to $80,000 per QALY and sintilimab plus a bevacizumab biosimilar was an affordable option at a WTP threshold of higher than $80,000 per QALY. Due to China’s vast territory and abundant resources, GDP per capita varies greatly. We also calculated WTP values for different regions of China. For example, The WTP values of Beijing, Shanghai, Jiangsu, Guangdong, Hubei, Neimenggu, Anhui, Hunan, Jiangxi, Guizhou, Guangxi, Heilongjiang and Gansu were $80,053/QALY, $75,656/QALY, $59,768/QALY, $42,964/QALY, $37,698/QALY, $37,132/QALY, $30,646/QALY, $30,167/QALY, $28,513/QALY, $22,114/QALY, $21,740/QALY, $20,329/QALY and $17,804/QALY, respectively (43). Surprisingly, tislelizumab is the best strategy of choice in underdeveloped and relatively developed regions of China. In most developed regions, camrelizumab plus rivoceranib is regarded as the best choice. Only in Beijing, sintilimab plus a bevacizumab biosimilar is an optional choice. Therefore, the different economic factors of different regions should be taken into account when approving novel medicines for clinical use. Currently, the drugs used in the first-line treatment of liver cancer have certain clinical benefits and may be used on a large scale, but their economic toxicity still exists. Economic toxicity can bankrupt patients with high treatment costs, cause cancer patients to stop treatment, and even lead to poor patient outcomes in China (34). In the CSCO guidelines version 2022, sorafenib, lenvatinib, donafenib, sintilimab plus a bevacizumab biosimilar, and camrelizumab plus rivoceranib are recommended first-line strategies, with a wide range of costs (23). Thus, our results can be used to find a reasonable balance between the price of new drugs and their clinical efficacy, inform national regulatory agencies when making healthcare decisions, and make a significant contribution to adequately address economic toxicity.

There are some limitations in our study. Firstly, when using the network meta-analysis method to compare first-line treatment regimens indirectly, we assumed that the included studies did not differ in patient characteristics and summarized the chemotherapy groups, and selected a fixed-effect model. However, it is a difference that we cannot eliminate. For instance, the ORIENT-32 trial recruited participants from the Chinese population and the IMbrave150 trial and LEAP-002 trial recruited globally. Secondly, considering that there were multiple survival curves of sorafenib, we pooled and reconstructed the survival curves according to the original survival data of the sorafenib group in each clinical trial. Thirdly, we inferred the long-term survival benefit in terms of the short-term survival data of each experiment, which will change with the change of long-term follow-up. This is an inevitable limitation in our model. Therefore, it is necessary to verify and evaluate the concordance of these health outcomes in a model with real-world data. In addition, we only considered the occurrence of more than 5% of grade 3 or higher adverse events, which may underestimate the cost of adverse events. Nevertheless, the cost and disutility of AEs were not the significant factors influencing the results

## 5 Conclusion

Briefly, sintilimab plus a bevacizumab biosimilar and camrelizumab plus rivoceranib showed the best efficacy in prolonging OS and PFS compared with sorafenib, respectively. What’s more, we found that tislelizumab is the most cost-effective first-line treatment strategy for unresectable HCC in China at the WTP of $37,653 QALY. In economically developed areas of China, camrelizumab plus rivoceranib is also a recommended cost-effective treatment strategy. The results could help clinicians select the most appropriate drugs for their patients and set reimbursement policies.

## Data availability statement

The original contributions presented in the study are included in the article/**Supplementary Material**. Further inquiries can be directed to the corresponding author.

## Ethics statement

This article is based on previously conducted studies and does not contain any new studies with human participants or animals performed by any of the authors, it does not require the approval of the independent ethics committee.

## Author contributions

KL, YZ, and HZ designed the experiment. YZ, and KL performed the experiments. YZ and KL analyzed the data. HZ contributed analysis tools and funding. YZ, KL, and HZ wrote the manuscript. KL and YZ contributed equally. All authors contributed to the article and approved the submitted version.
