# Supplementary material for: Immunotherapy or targeted therapy as the first-line strategies for unresectable hepatocellular carcinoma: A network meta-analysis and cost-effectiveness analysis
Source: Front Immunol. 2023 Jan 11;13:1103055. doi: 10.3389/fimmu.2022.1103055 (PMC9874298; doi:10.3389/fimmu.2022.1103055)
Supplement: Supplementary file 1 [file DataSheet_1.pdf]

eFigure 1. Model Structure for Cost-effectiveness Analysis.

eFigure 2. Kaplan-Meier Curve Fitting and Extrapolation.

eFigure 3. Study Selection

eFigure 4. Model of Network Meta-analysis.

eFigure 5. The Forest Plots of All-Grade (A) and Grade 3 or Higher AEs (B) in the Comparisons of Seven ICI Regimens Versus Chemotherapy Treatment.

eFigure 6. Probability Sensitivity Analysis Scatter Plot.

eTable 1. PRISMA NMA Checklist.

eTable 2. Search Strategy.

eTable 3. CHEERS Checklist.

eTable 4. Drug Dose and Cost.

eTable 5. Summary of Statistical Goodness-of-fit of Kaplan-Meier Curve.

eTable 6. Characteristics of RCTs Included in the Study.

eTable 7. Risk of Bias Summary.

eTable 8. Pairwise Comparison of ICER ($/QALY)

eFigure 1. Model Structure for Cost-effectiveness Analysis.

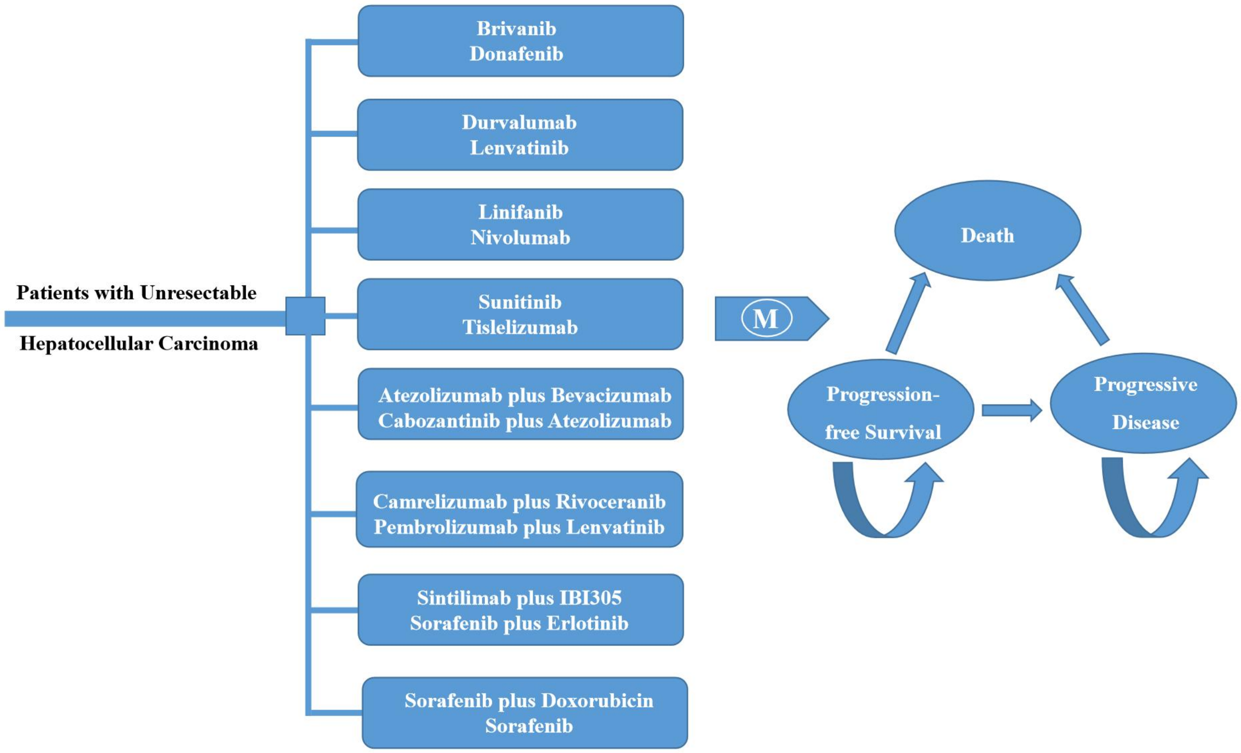

Abbreviation: IBI305, bevacizumab biosimilar; M, Markov.

eFigure 2. Kaplan-Meier Curve Fitting and Extrapolation.

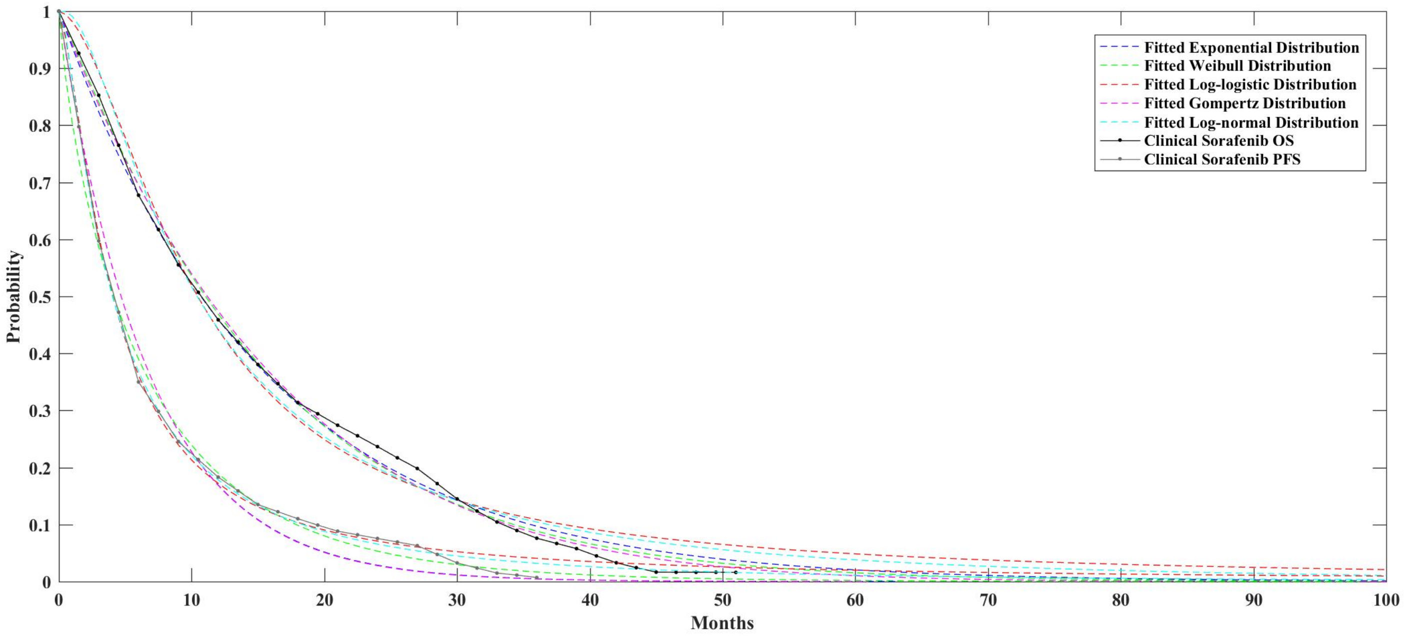

Abbreviation: OS, overall survival; PFS, progression-free survival.

eFigure 3. Study Selection.

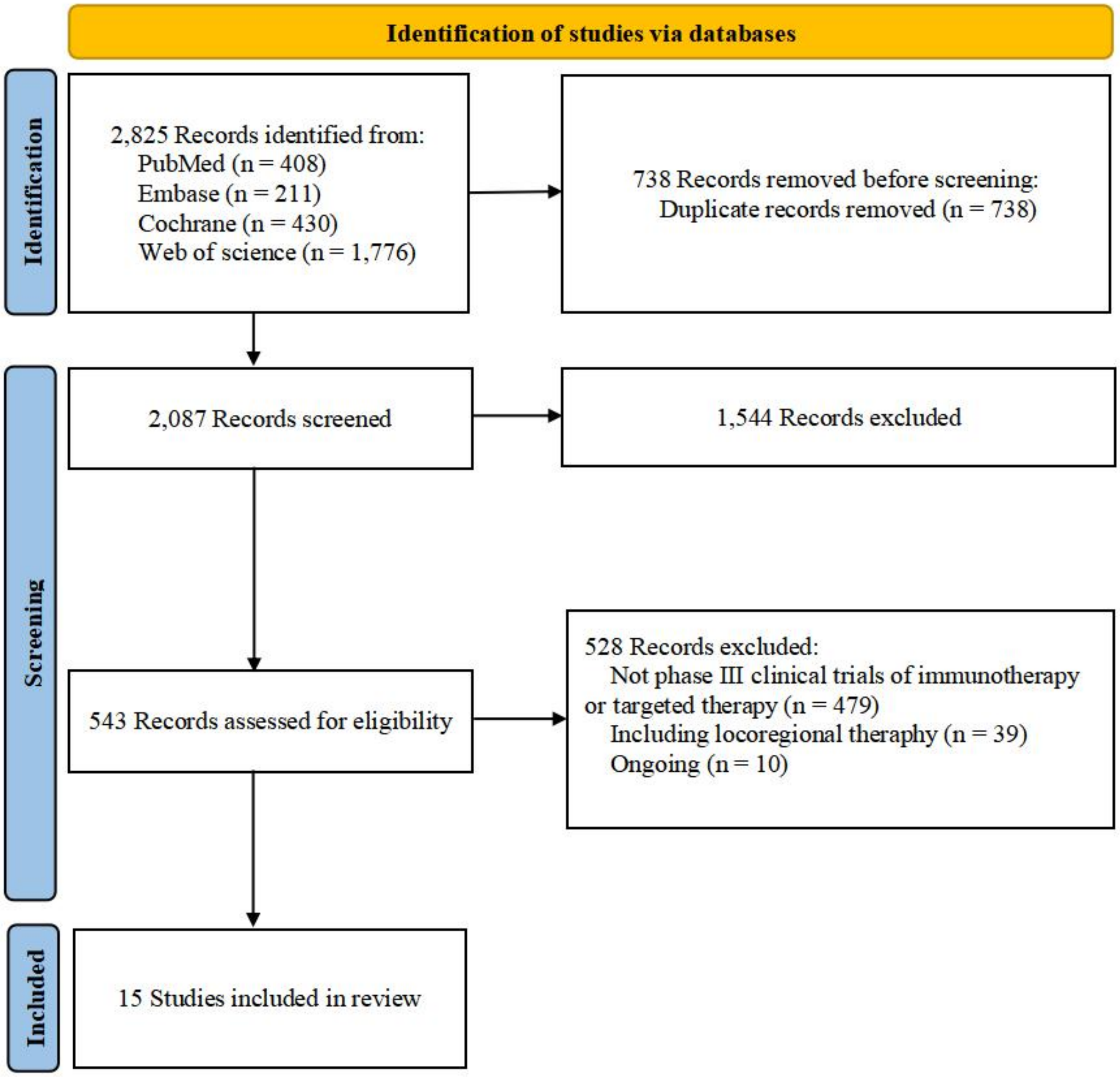

eFigure 4. Model of Network Meta-analysis.

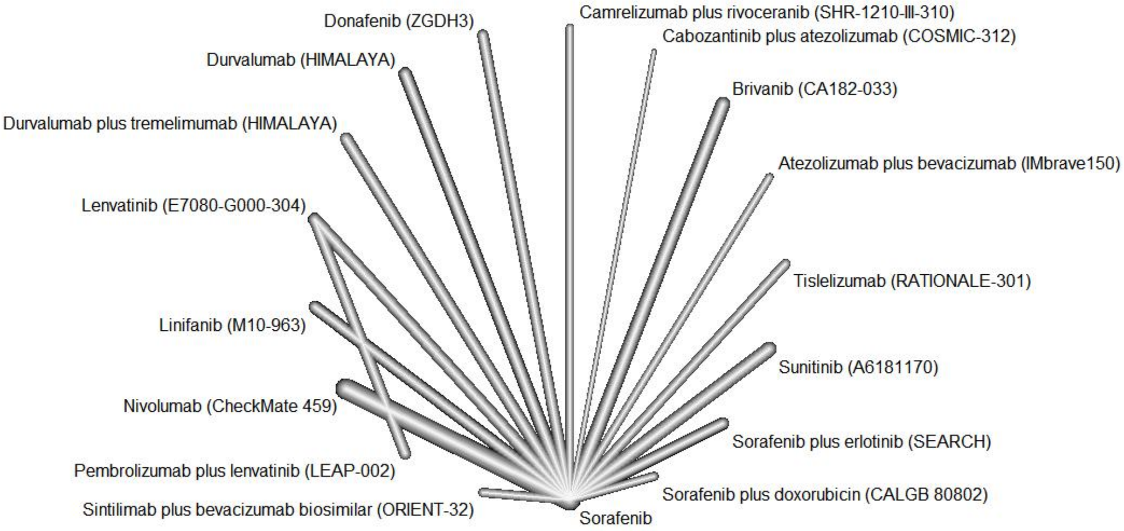

eFigure 5. The Forest Plots of all-grade (A) and Grade 3 or Higher AEs (B) in the Comparisons of Systemic Therapies Versus Sorafenib Treatment.

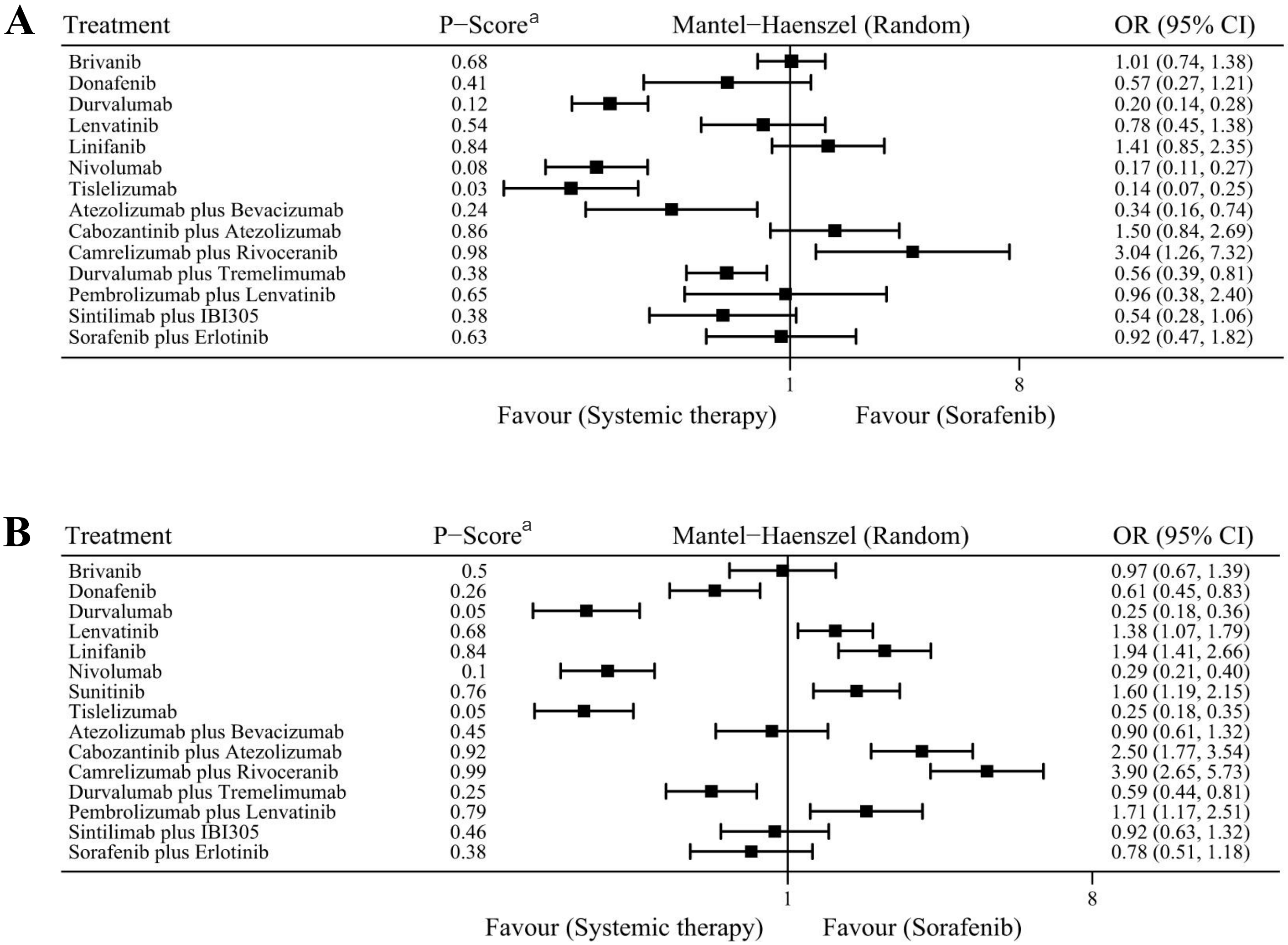

[a] Ranking of events causing more serious adverse events.

Abbreviation: OR, odds ratio; IBI305, bevacizumab biosimilar.

eFigure 6. Probability Sensitivity Analysis Scatter Plot.

A

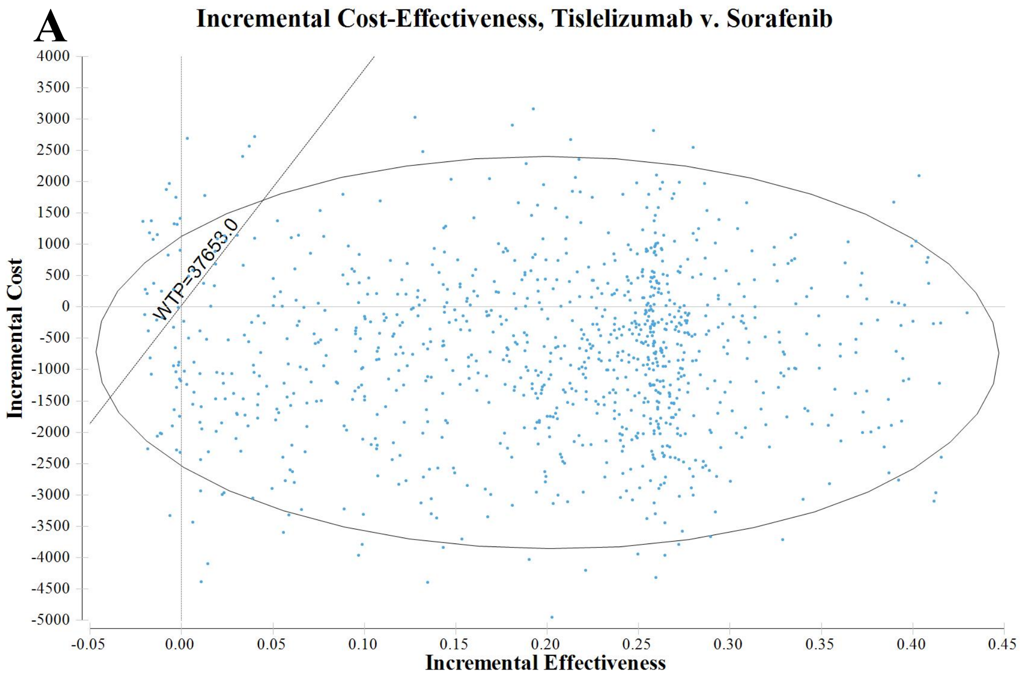

B

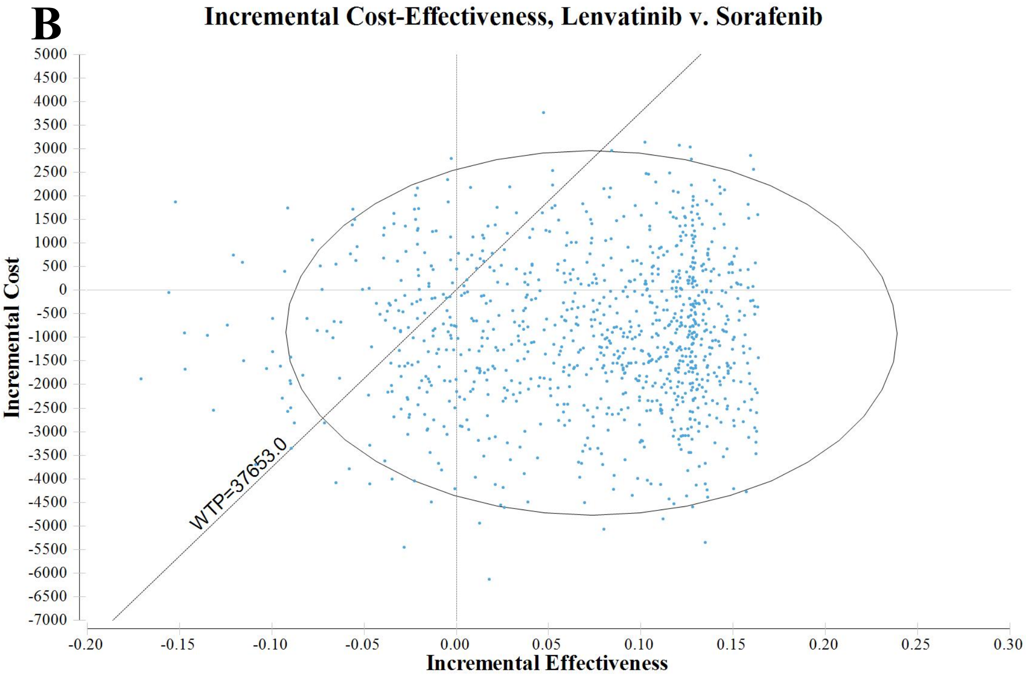

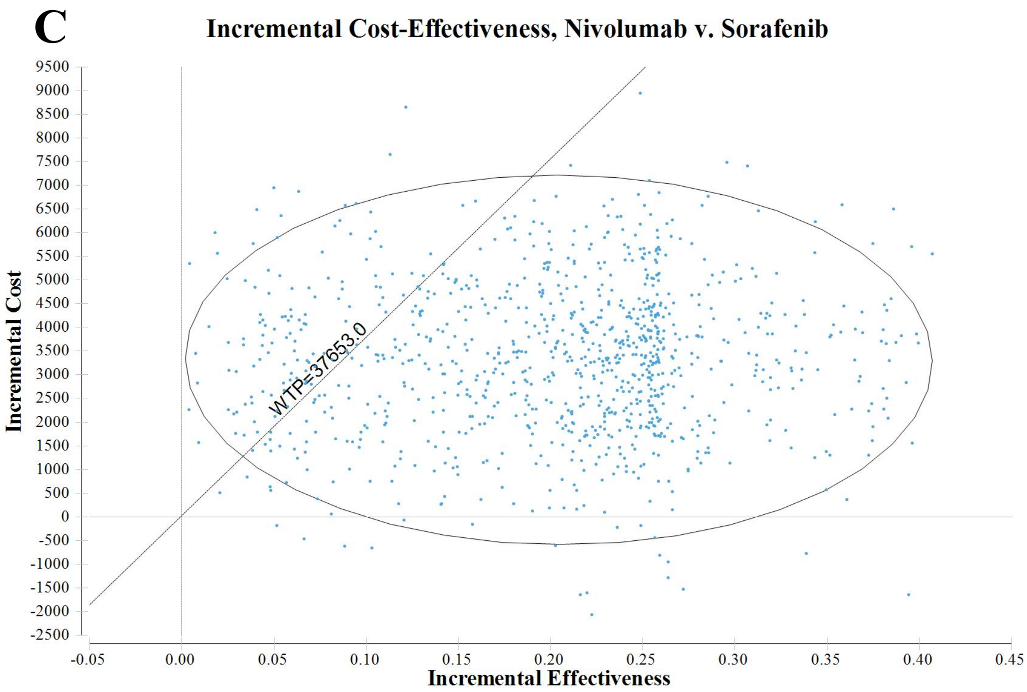
C
Incremental Cost-Effectiveness, Nivolumab v. Sorafenib
Incremental Cost
Incremental Effectiveness
WTP=37653.0

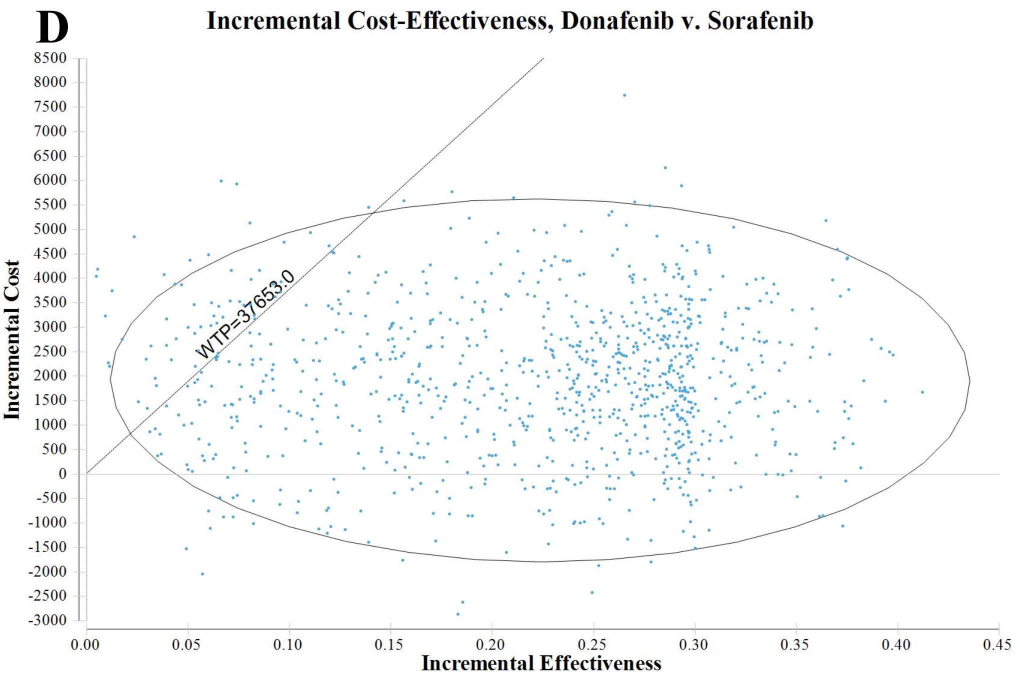
D
Incremental Cost-Effectiveness, Donafenib v. Sorafenib
Incremental Cost
Incremental Effectiveness
WTP=37653.0

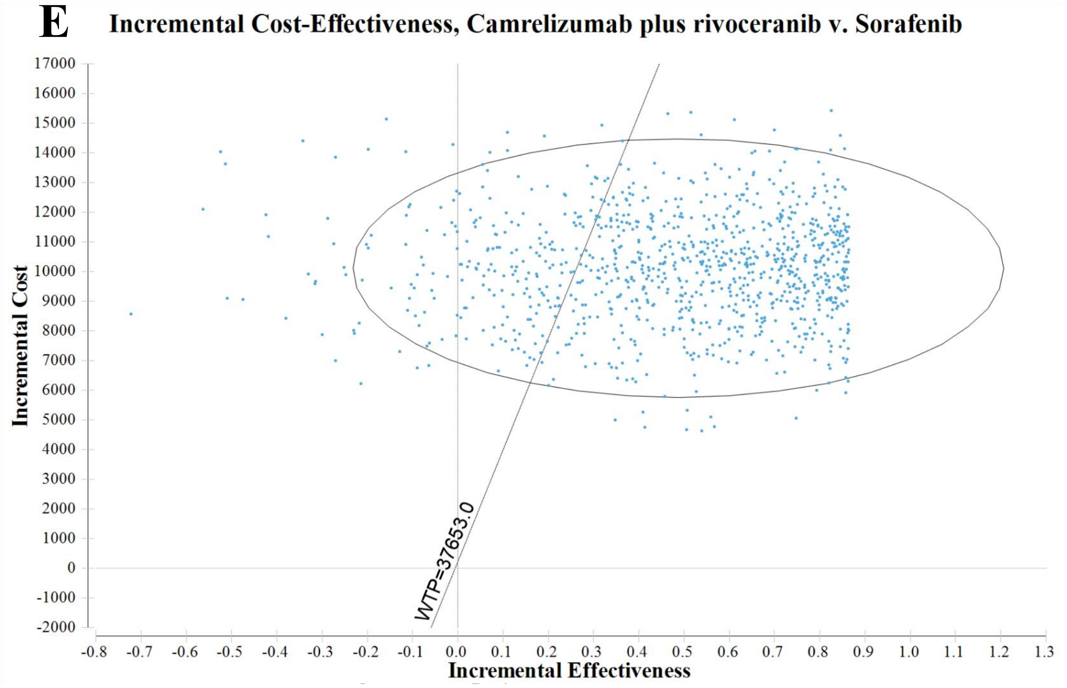
E
Incremental Cost-Effectiveness, Camrelizumab plus rivoceranib v. Sorafenib
Incremental Cost
Incremental Effectiveness
WTP=37653.0
17000
16000
15000
14000
13000
12000
11000
10000
9000
8000
7000
6000
5000
4000
3000
2000
1000
0
-1000
-2000
-0.8
-0.7
-0.6
-0.5
-0.4
-0.3
-0.2
-0.1
0.0
0.1
0.2
0.3
0.4
0.5
0.6
0.7
0.8
0.9
1.0
1.1
1.2
1.3

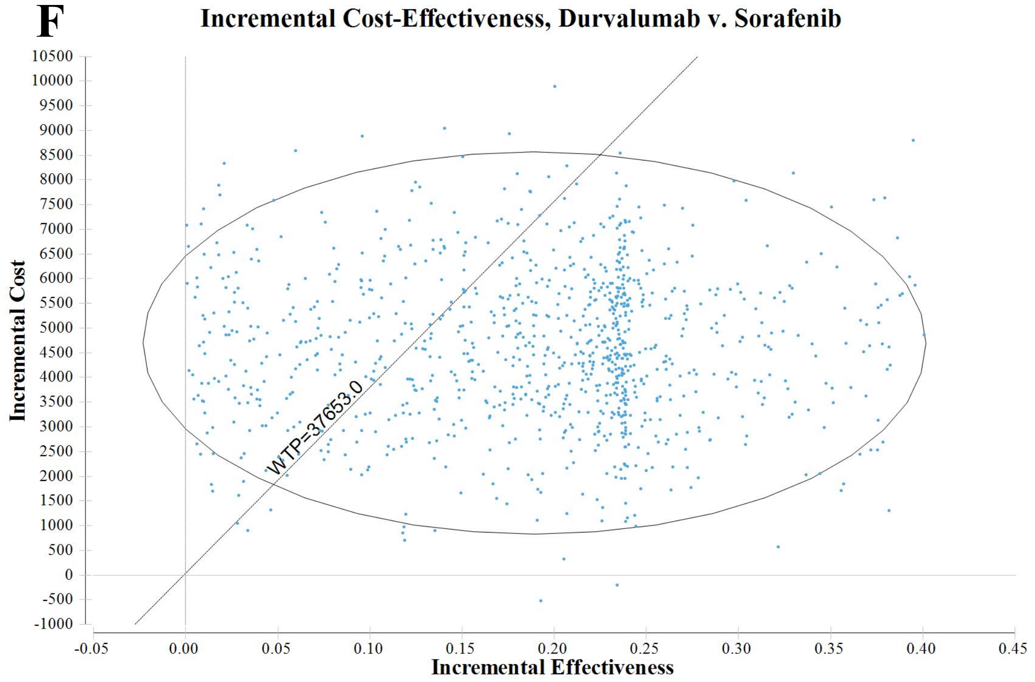
F
Incremental Cost-Effectiveness, Durvalumab v. Sorafenib
Incremental Cost
Incremental Effectiveness
WTP=37653.0
10500
10000
9500
9000
8500
8000
7500
7000
6500
6000
5500
5000
4500
4000
3500
3000
2500
2000
1500
1000
500
0
-500
-1000
-0.05
0.00
0.05
0.10
0.15
0.20
0.25
0.30
0.35
0.40
0.45

G Incremental Cost-Effectiveness, Sintilimab plus IBI305 v. Sorafenib

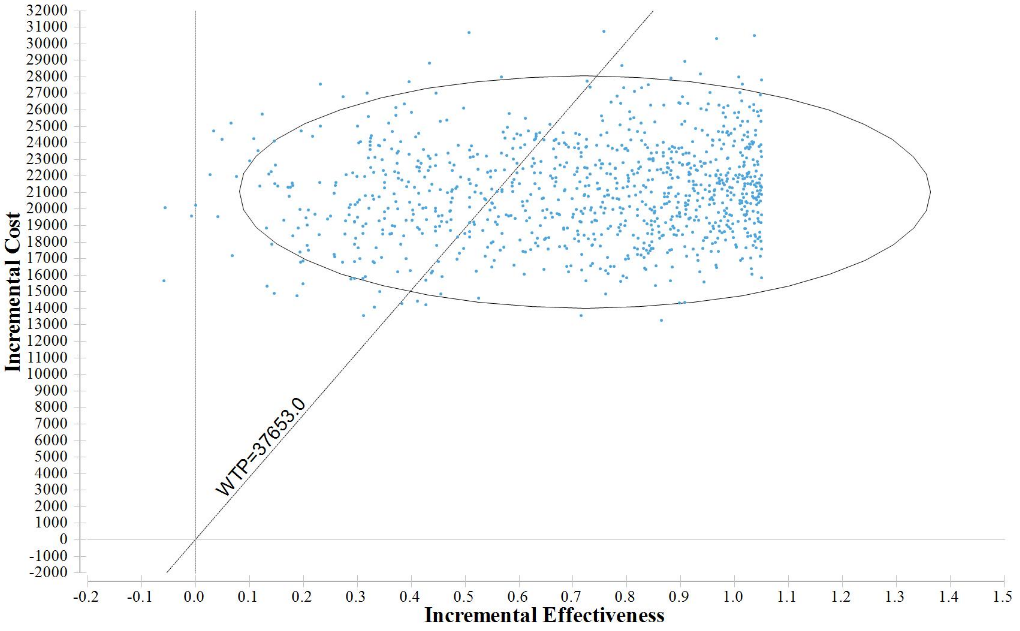

H Incremental Cost-Effectiveness, Pembrolizumab plus lenvatinib v. Sorafenib

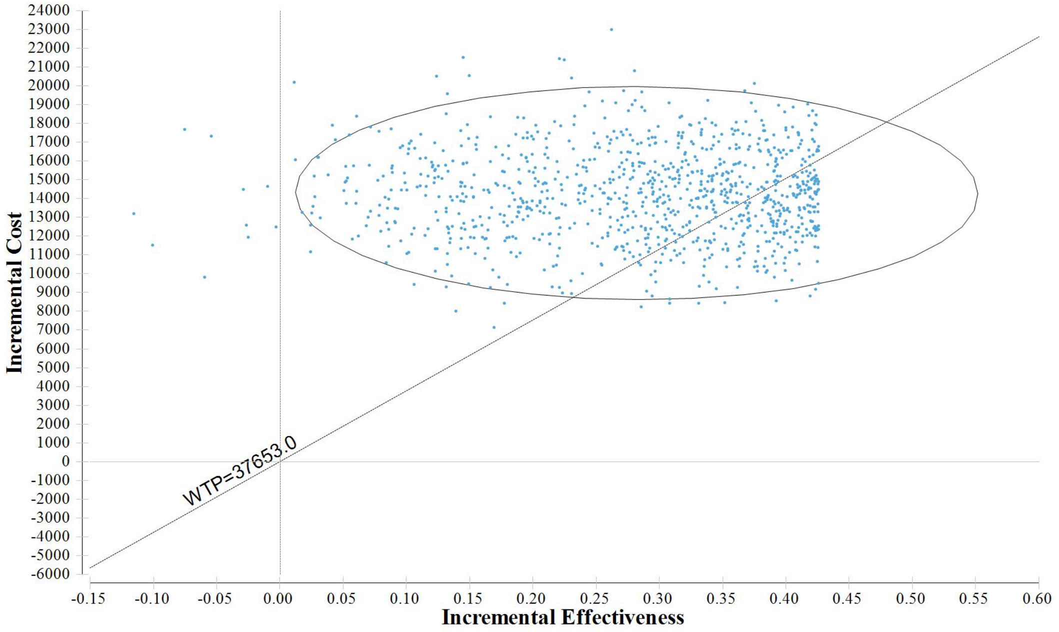

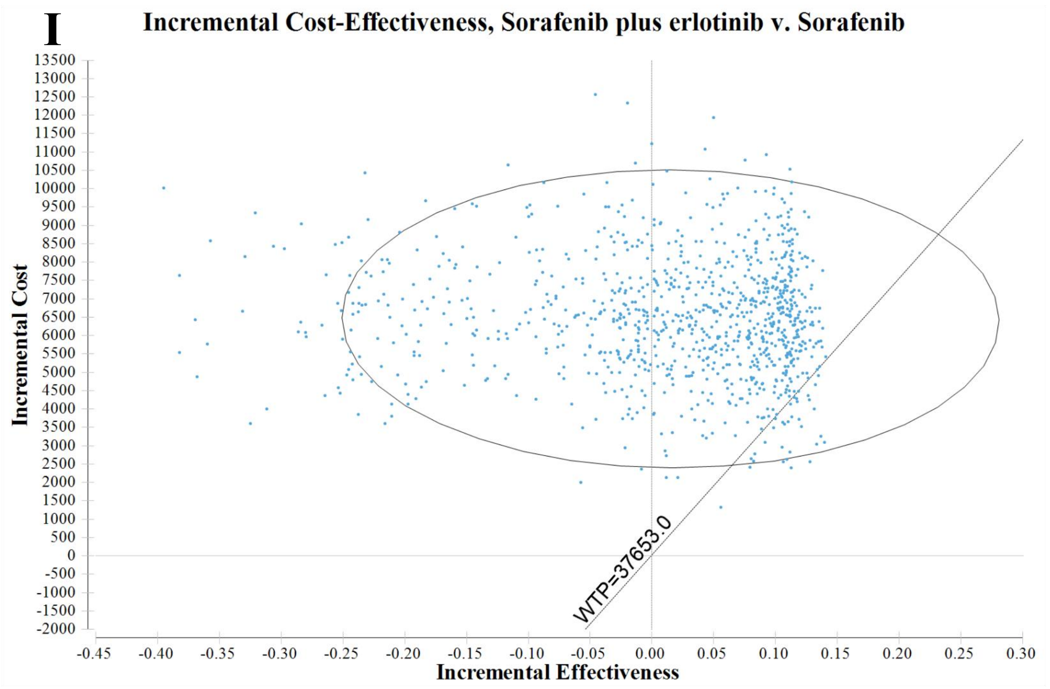
I
Incremental Cost-Effectiveness, Sorafenib plus erlotinib v. Sorafenib
Incremental Cost
Incremental Effectiveness
WTP=37653.0

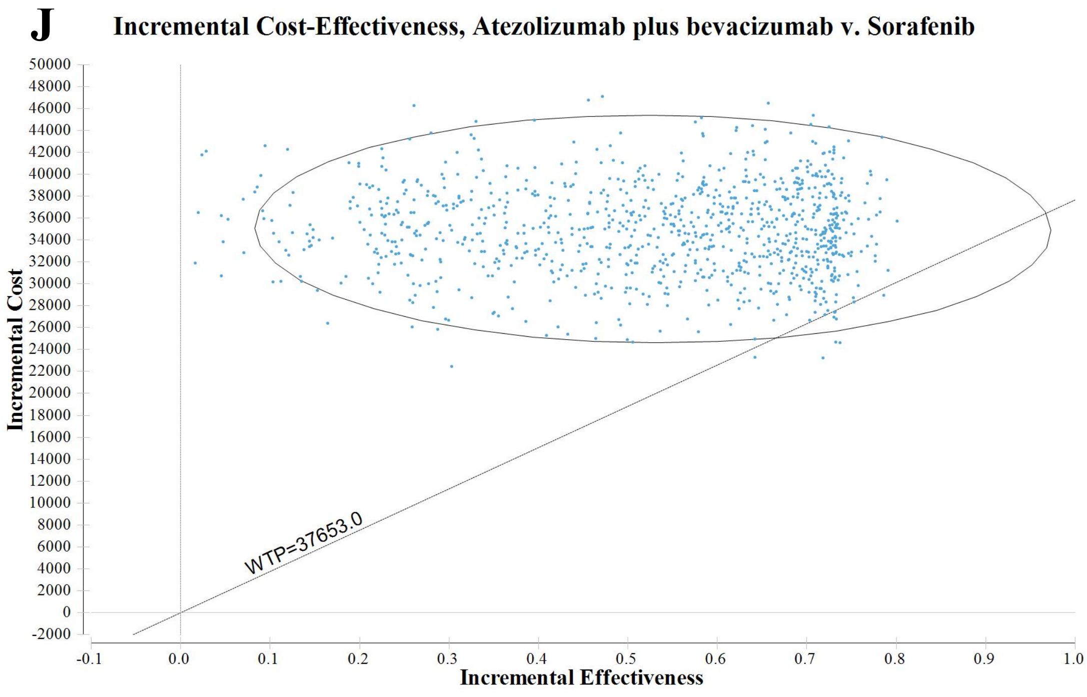
J
Incremental Cost-Effectiveness, Atezolizumab plus bevacizumab v. Sorafenib
Incremental Cost
Incremental Effectiveness
WTP=37653.0

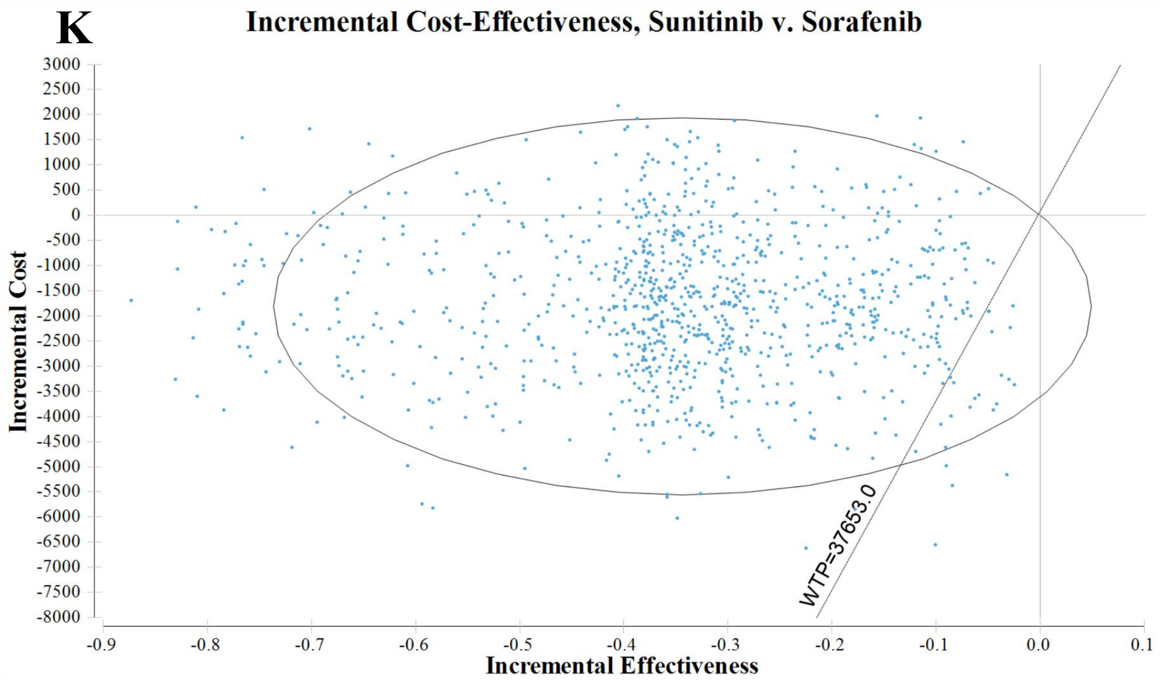
K
Incremental Cost-Effectiveness, Sunitinib v. Sorafenib
Incremental Cost
Incremental Effectiveness
WTP=37653.0
3000
2500
2000
1500
1000
500
0
-500
-1000
-1500
-2000
-2500
-3000
-3500
-4000
-4500
-5000
-5500
-6000
-6500
-7000
-7500
-8000
-0.9
-0.8
-0.7
-0.6
-0.5
-0.4
-0.3
-0.2
-0.1
0.0
0.1

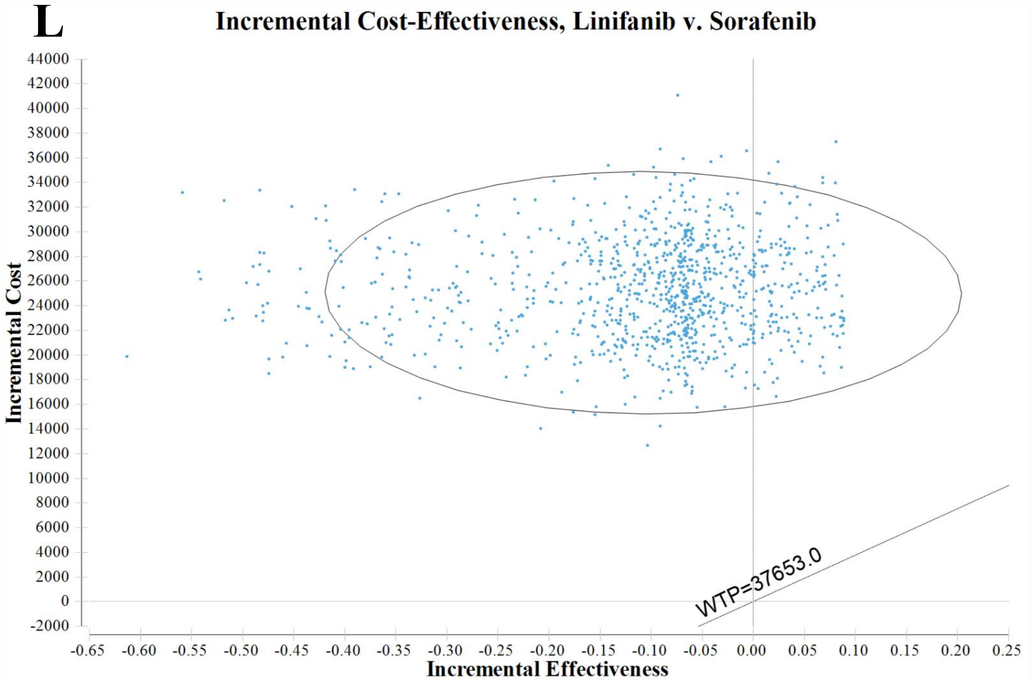
L
Incremental Cost-Effectiveness, Linifanib v. Sorafenib
Incremental Cost
Incremental Effectiveness
WTP=37653.0
44000
42000
40000
38000
36000
34000
32000
30000
28000
26000
24000
22000
20000
18000
16000
14000
12000
10000
8000
6000
4000
2000
0
-2000
-0.65
-0.60
-0.55
-0.50
-0.45
-0.40
-0.35
-0.30
-0.25
-0.20
-0.15
-0.10
-0.05
0.00
0.05
0.10
0.15
0.20
0.25

M **Incremental Cost-Effectiveness, Brivanib v. Sorafenib**

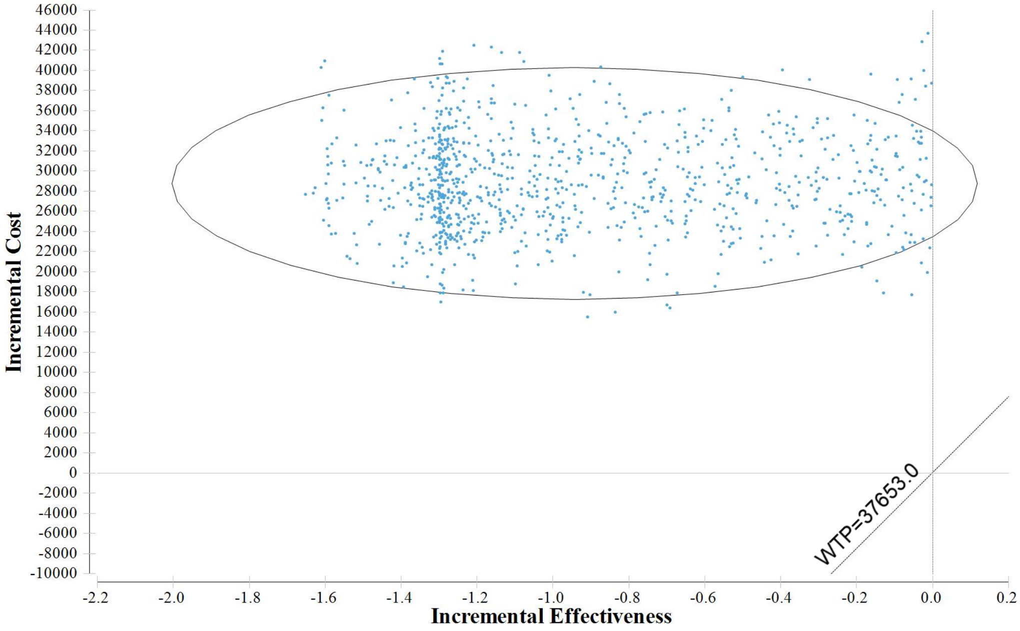

N **Incremental Cost-Effectiveness, Cabozantinib plus atezolizumab v. Sorafenib**

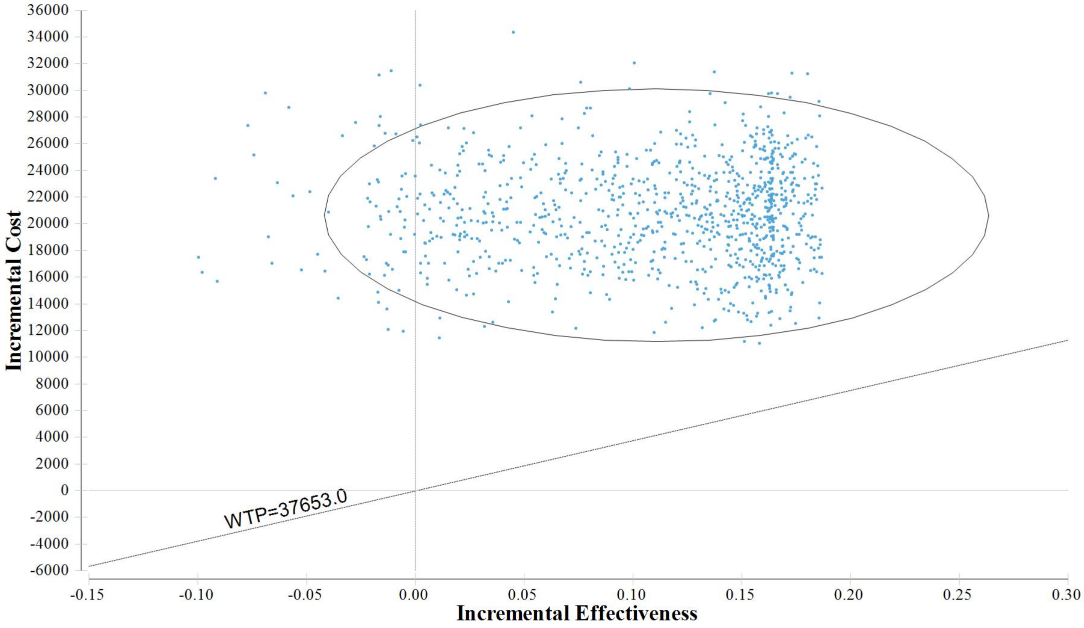

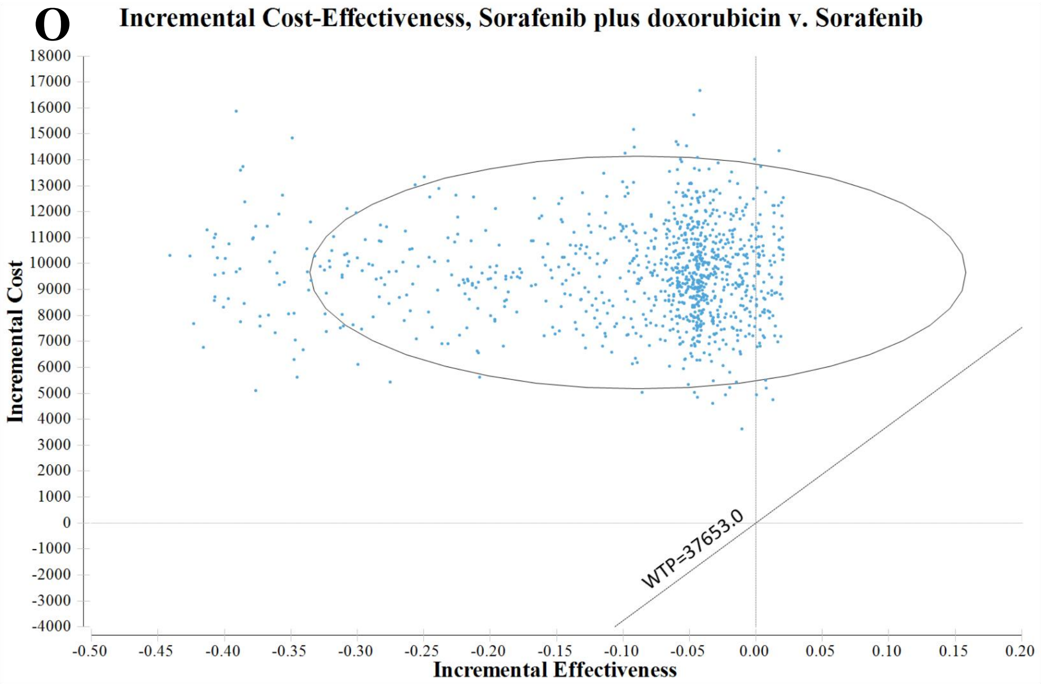

Abbreviation: WTP, willingness-to-pay; IBI305, bevacizumab biosimilar.

Probability Sensitivity analyses of tislelizumab (A), lenvatinib (B), nivolumab (C), donafenib (D), camrelizumab plus rivoceranib (E), durvalumab (F), sintilimab plus IBI305(G), pembrolizumab plus lenvatinib (H), sorafenib plus erlotinib (I), atezolizumab plus bevacizumab (J), sunitinib (K), linifanib (L), brivanib (M), cabozantinib plus atezolizumab (N), sorafenib plus doxorubicin (O) in comparison with sorafenib.

Each point in the diagram represents a simulation result of 10,000 Monte Carlo simulation. Ellipse represent the 95% CI and dotted line represent WTP ($37,653/QALY). Points to the right of the dotted line are considered cost-effective.

eTable 1. PRISMA NMA Checklist.

Section/Topic	Item #	Checklist Item	Reported on Page #
**TITLE**			
Title	1	Identify the report as a systematic review *incorporating a network meta-analysis (or related form of meta-analysis).*	1
**ABSTRACT**			
Structured summary	2	Provide a structured summary including, as applicable:<br>**Background:** main objectives<br>**Methods:** data sources; study eligibility criteria, participants, and interventions; study appraisal; and *synthesis methods, such as network meta-analysis.*<br>**Results:** number of studies and participants identified; summary estimates with corresponding confidence/credible intervals; *treatment rankings may also be discussed. Authors may choose to summarize pairwise comparisons against a chosen treatment included in their analyses for brevity.*<br>**Discussion/Conclusions:** limitations; conclusions and implications of findings.<br>**Other:** primary source of funding; systematic review registration number with registry name.	2
**INTRODUCTION**			
Rationale	3	Describe the rationale for the review in the context of what is already known*, including mention of why a network meta-analysis has been conducted.*	3-4
Objectives	4	Provide an explicit statement of questions being addressed, with reference to participants, interventions, comparisons, outcomes, and study design (PICOS).	4
**METHODS**			
Protocol and registration	5	Indicate whether a review protocol exists and if and where it can be accessed (e.g., Web address); and, if available, provide registration information, including registration number.	Not applicable
Eligibility criteria	6	Specify study characteristics (e.g., PICOS, length of follow-up) and report characteristics (e.g., years considered, language, publication status) used as criteria for eligibility, giving rationale. *Clearly describe eligible treatments included in the treatment network, and note whether any have been clustered or merged into the same node (with justification).*	5<br>eTable 6
Information sources	7	Describe all information sources (e.g., databases with dates of coverage, contact with study authors to identify additional studies) in the search and date last searched.	5
Search	8	Present full electronic search strategy for at least one database, including any limits used, such that it could be repeated.	5<br>eTable 2
Study selection	9	State the process for selecting studies (i.e., screening, eligibility, included in systematic review, and, if applicable,	eFigure 3

		included in the meta-analysis).	
Data collection process	10	Describe method of data extraction from reports (e.g., piloted forms, independently, in duplicate) and any processes for obtaining and confirming data from investigators.	5<br>eFigure 3<br>eTable 6
Data items	11	List and define all variables for which data were sought (e.g., PICOS, funding sources) and any assumptions and simplifications made.	eTable 6<br>Table 1
**Geometry of the network**	**S1**	Describe methods used to explore the geometry of the treatment network under study and potential biases related to it. This should include how the evidence base has been graphically summarized for presentation, and what characteristics were compiled and used to describe the evidence base to readers.	eFigure 4
Risk of bias within individual studies	12	Describe methods used for assessing risk of bias of individual studies (including specification of whether this was done at the study or outcome level), and how this information is to be used in any data synthesis.	eTable 7
Summary measures	13	State the principal summary measures (e.g., risk ratio, difference in means). *Also describe the use of additional summary measures assessed, such as treatment rankings and surface under the cumulative ranking curve (SUCRA) values, as well as modified approaches used to present summary findings from meta-analyses.*	5<br>Figure 1<br>Figure 2
Planned methods of analysis	14	Describe the methods of handling data and combining results of studies for each network meta-analysis. This should include, but not be limited to:<br>• *Handling of multi-arm trials;*<br>• *Selection of variance structure;*<br>• *Selection of prior distributions in Bayesian analyses; and*<br>•*Assessment of model fit.*	5
**Assessment of Inconsistency**	**S2**	Describe the statistical methods used to evaluate the agreement of direct and indirect evidence in the treatment network(s) studied. Describe efforts taken to address its presence when found.	5
Risk of bias across studies	15	Specify any assessment of risk of bias that may affect the cumulative evidence (e.g., publication bias, selective reporting within studies).	eTable 7
Additional analyses	16	Describe methods of additional analyses if done, indicating which were pre-specified. This may include, but not be limited to, the following:<br>• Sensitivity or subgroup analyses; • Meta-regression analyses;<br>• *Alternative formulations of the treatment network; and* • *Use of alternative prior distributions for Bayesian analyses (if applicable).*	5

**RESULTS†**			
Study selection	17	Give numbers of studies screened, assessed for eligibility, and included in the review, with reasons for exclusions at each stage, ideally with a flow diagram.	7-8<br>eFigure 3
**Presentation of network structure**	**S3**	Provide a network graph of the included studies to enable visualization of the geometry of the treatment network.	eFigure 4
**Summary of network geometry**	**S4**	Provide a brief overview of characteristics of the treatment network. This may include commentary on the abundance of trials and randomized patients for the different interventions and pairwise comparisons in the network, gaps of evidence in the treatment network, and potential biases reflected by the network structure.	8<br>eFigure 4
Study characteristics	18	For each study, present characteristics for which data were extracted (e.g., study size, PICOS, follow-up period) and provide the citations.	8<br>eTable 6
Risk of bias within studies	19	Present data on risk of bias of each study and, if available, any outcome level assessment.	8<br>eTable 7
Results of individual studies	20	For all outcomes considered (benefits or harms), present, for each study: 1) simple summary data for each intervention group, and 2) effect estimates and confidence intervals. *Modified approaches may be needed to deal with information from larger networks.*	8<br>eTable 6
Synthesis of results	21	Present results of each meta-analysis done, including confidence/credible intervals. *In larger networks, authors may focus on comparisons versus a particular comparator (e.g. placebo or standard care), with full findings presented in an appendix. League tables and forest plots may be considered to summarize pairwise comparisons.* If additional summary measures were explored (such as treatment rankings), these should also be presented.	8<br>Table 1<br>Table 2
**Exploration for inconsistency**	**S5**	Describe results from investigations of inconsistency. This may include such information as measures of model fit to compare consistency and inconsistency models, *P* values from statistical tests, or summary of inconsistency estimates from different parts of the treatment network.	Not applicable
Risk of bias across studies	22	Present results of any assessment of risk of bias across studies for the evidence base being studied.	Not applicable
Results of additional analyses	23	Give results of additional analyses, if done (e.g., sensitivity or subgroup analyses, meta-regression analyses*, alternative network geometries studied, alternative choice of prior distributions for Bayesian analyses,* and so forth).	8-11<br>eFigure 4
**DISCUSSION**			
Summary of evidence	24	Summarize the main findings, including the strength of evidence for each main outcome; consider their relevance to key groups (e.g., healthcare providers, users, and policy-makers).	10-12
Limitations	25	Discuss limitations at study and outcome level (e.g., risk of bias), and at review level (e.g., incomplete retrieval of	13

		identified research, reporting bias). *Comment on the validity of the assumptions, such as transitivity and consistency. Comment on any concerns regarding network geometry (e.g., avoidance of certain comparisons).*	
Conclusions	26	Provide a general interpretation of the results in the context of other evidence, and implications for future research.	14
**FUNDING**			
Funding	27	Describe sources of funding for the systematic review and other support (e.g., supply of data); role of funders for the systematic review. This should also include information regarding whether funding has been received from manufacturers of treatments in the network and/or whether some of the authors are content experts with professional conflicts of interest that could affect use of treatments in the network.	14

Abbreviation: PICOS = population, intervention, comparators, outcomes, study design.

* Text in italics indicates wording specific to reporting of network meta-analyses that has been added to guidance from the PRISMA statement.

† Authors may wish to plan for use of appendices to present all relevant information in full detail for items in this section.

Reference:

Hutton B, Salanti G, Caldwell DM et al. The PRISMA extension statement for reporting of systematic reviews incorporating network meta-analyses of health care interventions: checklist and explanations. Ann Intern Med 2015; 162: 777-784.

eTable 2. Search Strategy.

**PubMed**	
**(1)**	"nivolumab"[Title/Abstract] OR "pembrolizumab"[Title/Abstract] OR "ipilimumab" [Title/Abstract] OR "atezolizumab"[Title/Abstract] OR "camrelizumab"[Title/Abstract] OR "cemiplimab"[Title/Abstract] OR "durvalumab"[Title/Abstract] OR "toripalimab"[Title/Abstract] OR "tislelizumab"[Title/Abstract] OR "PD-1"[Title/Abstract] OR "PD-L1"[Title/Abstract] OR "anti-PD-1"[Title/Abstract] OR "anti-PD-L1"[Title/Abstract] OR "immune checkpoint inhibitor"[Title/Abstract] OR "ICIs "[Title/Abstract] OR "programmed cell death 1 receptor/antagonists and inhibitors"[Mesh] OR "programmed cell death 1 receptor antagonists and inhibitors"[Title/Abstract] OR "programmed cell death 1 receptor antagonist"[Title/Abstract] OR "programmed cell death 1 receptor inhibitor"[Title/Abstract] OR "immunotherapy"[Title/Abstract] OR "immune checkpoint inhibitors"[Mesh] OR "sorafenib"[Title/Abstract] OR "sunitinib"[Title/Abstract] OR " linifanib"[Title/Abstract] OR " lenvatinib"[Title/Abstract] OR "donafenib"[Title/Abstract] OR "bevacizumab"[Title/Abstract] OR "targeted therapy"[Title/Abstract] OR "molecular targeted therapy"[Mesh]
**(2)**	"unresectable hepatocellular carcinoma"[Title/Abstract] OR "advanced hepatocellular carcinoma"[Title/Abstract] OR "metastatic hepatocellular carcinoma"[Title/Abstract]
**(3)**	"clinical trials as topic"[Mesh] OR "clinical trial"[Publication Type] OR "clinical trials"[Title/Abstract] OR "phase III"[Title/Abstract] OR "phase 3"[Title/Abstract]
**(4)**	("2010/01/01"[Date - Publication]: "2022/09/20"[Date - Publication])
**(5)**	(1) AND (2) AND (3) AND (4)
**(6)**	"review"[Article type] OR "meta"[Title] OR "meta-analysis"[Title] OR "protocol"[Title] OR "cost-effectiveness"[Title]
**(7)**	(5) NOT (6)
**Embase**	
**(1)**	(nivolumab OR pembrolizumab OR ipilimumab OR atezolizumab OR camrelizumab OR cemiplimab OR durvalumab OR toripalimab OR tislelizumab OR PD-1 OR PD-L1 OR anti-PD-1 OR anti-PD-L1 OR 'immune checkpoint inhibitor' OR 'ICIs' OR 'programmed cell death 1 receptor/antagonists and inhibitors' OR 'programmed cell death 1 receptor antagonists and inhibitors' OR 'programmed cell death 1 receptor antagonist' OR 'programmed cell death 1 receptor inhibitor' OR 'immunotherapy' OR 'immune checkpoint inhibitors' OR sorafenib OR sunitinib OR linifanib OR lenvatinib OR donafenib OR bevacizumab OR 'targeted therapy' OR 'molecular targeted therapy'):ti,ab,kw
**(2)**	('unresectable hepatocellular carcinoma' OR 'advanced hepatocellular carcinoma' OR 'metastatic hepatocellular carcinoma'):ti,ab,kw

**(3)**	trial/exp OR 'clinical trials'/exp OR 'phase 3 clinical trial'/exp OR 'phase III clinical trial'/exp
**(4)**	[article]/lim OR [article in press]/lim
**(5)**	[humans]/lim
**(6)**	[1-1-2010]/sd NOT [20-9-2022]/sd
**(7)**	(1) AND (2) AND (3) AND (4) AND (5) AND (6)
**Cochrane**	
**(1)**	(nivolumab OR pembrolizumab OR ipilimumab OR atezolizumab OR camrelizumab OR cemiplimab OR durvalumab OR toripalimab OR tislelizumab OR PD-1 OR PD-L1 OR anti-PD-1 OR anti-PD-L1 OR 'immune checkpoint inhibitor' OR 'ICIs' OR 'programmed cell death 1 receptor antagonists and inhibitors' OR 'programmed cell death 1 receptor antagonist' OR 'programmed cell death 1 receptor inhibitor' OR 'immunotherapy' OR 'immune checkpoint inhibitors' OR sorafenib OR sunitinib OR linifanib OR lenvatinib OR donafenib OR bevacizumab OR 'targeted therapy' OR 'molecular targeted therapy'):ti,ab,kw
**(2)**	('unresectable hepatocellular carcinoma' OR 'advanced hepatocellular carcinoma' OR 'metastatic hepatocellular carcinoma'):ti,ab,kw
**(3)**	(trial OR clinical trials OR 'phase 3 clinical trial' OR 'phase III clinical trial'):ti,ab,kw
**(4)**	(review OR meta OR meta-analysis OR protocol OR cost-effectivenessl OR conference):ti,ab,kw
**(5)**	(1) AND (2) AND (3) NOT (4) (custom range: 2010-01-01 to 2022-09-20)
**Web of science**	
**(1)**	TS=(nivolumab OR pembrolizumab OR ipilimumab OR atezolizumab OR camrelizumab OR cemiplimab OR durvalumab OR toripalimab OR tislelizumab OR PD-1 OR PD-L1 OR anti-PD-1 OR anti-PD-L1 OR 'immune checkpoint inhibitor' OR 'ICIs' OR 'programmed cell death 1 receptor/antagonists and inhibitors' OR 'programmed cell death 1 receptor antagonists and inhibitors' OR 'programmed cell death 1 receptor antagonist' OR 'programmed cell death 1 receptor inhibitor' OR 'immunotherapy' OR 'immune checkpoint inhibitors' OR sorafenib OR sunitinib OR linifanib OR lenvatinib OR donafenib OR bevacizumab OR 'targeted therapy' OR 'molecular targeted therapy')
**(2)**	TS=('unresectable hepatocellular carcinoma' OR 'advanced hepatocellular carcinoma' OR 'metastatic hepatocellular carcinoma')
**(3)**	TS=(trial OR clinical trials OR 'phase 3 clinical trial' OR 'phase III clinical trial')
**(4)**	TS=(conference OR review OR meta OR meta-analysis OR cost-effectiveness)
**(5)**	(1) AND (2) AND (3) NOT (4) AND (publication date: 2010-01-01 to 2022-09-20)

eTable 3. CHEERS Checklist.

Section/item	Item No	Recommendation	Reported on page No
**Title and abstract**			
Title	1	Identify the study as an economic evaluation or use more specific terms such as "cost-effectiveness analysis", and describe the interventions compared.	1
Abstract	2	Provide a structured summary of objectives, perspective, setting, methods (including study design and inputs), results (including base case and uncertainty analyses), and conclusions.	2
**Introduction**			
Background and objectives	3	Provide an explicit statement of the broader context for the study.	3-4
		Present the study question and its relevance for health policy or practice decisions.	
**Methods**			
Target population and subgroups	4	Describe characteristics of the base case population and subgroups analysed, including why they were chosen.	6
Setting and location	5	State relevant aspects of the system(s) in which the decision(s) need(s) to be made.	6
Study perspective	6	Describe the perspective of the study and relate this to the costs being evaluated.	6
Comparators	7	Describe the interventions or strategies being compared and state why they were chosen.	6
Time horizon	8	State the time horizon(s) over which costs and consequences are being evaluated and say why appropriate.	6
Discount rate	9	Report the choice of discount rate(s) used for costs and outcomes and say why appropriate.	6
Choice of health outcomes	10	Describe what outcomes were used as the measure(s) of benefit in the evaluation and their relevance for the type of analysis performed.	6
Measurement of effectiveness	11a	*Single study-based estimates:* Describe fully the design features of the single effectiveness study and why the single study was a sufficient source of clinical effectiveness data.	6,7

	11b	*Synthesis-based estimates:* Describe fully the methods used for identification of included studies and synthesis of clinical effectiveness data.	
Measurement and valuation of preference based outcomes	12	If applicable, describe the population and methods used to elicit preferences for outcomes.	6,7
Estimating resources and costs	13a	*Single study-based economic evaluation:* Describe approaches used to estimate resource use associated with the alternative interventions. Describe primary or secondary research methods for valuing each resource item in terms of its unit cost. Describe any adjustments made to approximate to opportunity costs.	6,7
	13b	*Model-based economic evaluation:* Describe approaches and data sources used to estimate resource use associated with model health states. Describe primary or secondary research methods for valuing each resource item in terms of its unit cost. Describe any adjustments made to approximate to opportunity costs.	
Currency, price date, and conversion	14	Report the dates of the estimated resource quantities and unit costs. Describe methods for adjusting estimated unit costs to the year of reported costs if necessary. Describe methods for converting costs into a common currency base and the exchange rate.	7
Choice of model	15	Describe and give reasons for the specific type of decision-analytical model used. Providing a figure to show model structure is strongly recommended.	6 and eFigure 2
Assumptions	16	Describe all structural or other assumptions underpinning the decision-analytical model.	6,7
Analytical methods	17	Describe all analytical methods supporting the evaluation. This could include methods for dealing with skewed, missing, or censored data; extrapolation methods; methods for pooling data; approaches to validate or make adjustments (such as half cycle corrections) to a model; and methods for handling population heterogeneity and uncertainty.	6,7
**Results**			

Study parameters	18	Report the values, ranges, references, and, if used, probability distributions for all parameters. Report reasons or sources for distributions used to represent uncertainty where appropriate. Providing a table to show the input values is strongly recommended.	8-10<br>Table 1
Incremental costs and outcomes	19	For each intervention, report mean values for the main categories of estimated costs and outcomes of interest, as well as mean differences between the comparator groups. If applicable, report incremental cost-effectiveness ratios.	8-10<br>Table 2
Characterising uncertainty	20a	*Single study-based economic evaluation:* Describe the effects of sampling uncertainty for the estimated incremental cost and incremental effectiveness parameters, together with the impact of methodological assumptions (such as discount rate, study perspective).	8-10<br>Table 3
	20b	*Model-based economic evaluation:* Describe the effects on the results of uncertainty for all input parameters, and uncertainty related to the structure of the model and assumptions.	
Characterising heterogeneity	21	If applicable, report differences in costs, outcomes, or cost-effectiveness that can be explained by variations between subgroups of patients with different baseline characteristics or other observed variability in effects that are not reducible by more information.	Not applicable
**Discussion**			
Study findings, limitations, generalisability, and current knowledge	22	Summarise key study findings and describe how they support the conclusions reached. Discuss limitations and the generalisability of the findings and how the findings fit with current knowledge.	10-14
**Other**			
Source of funding	23	Describe how the study was funded and the role of the funder in the identification, design, conduct, and reporting of the analysis. Describe other non-monetary sources of support.	14
Conflicts of interest	24	Describe any potential for conflict of interest of study contributors in accordance with journal policy. In the absence of a journal policy, we recommend authors comply with International Committee of Medical Journal Editors recommendations.	14

A good template page for CHEERS Checklist is as follows: http://www.ispor.org/TaskForces/EconomicPubGuidelines.asp.

Reference:

Husereau D, Drummond M, Petrou S, et al. Consolidated health economic evaluation reporting standards (CHEERS) — Explanation and elaboration: A report of the ISPOR health economic evaluations publication guidelines good reporting practices task force. Value Health 2013;16:231-50.

eTable 4. Drug dose and cost.

Drug	Dosage	Usage	Unit costs ($)
Brivanib	Brivanib, 800mg	Orally once daily	0.3205 per 1mg
Donafenib	Donafenib, 200mg	Orally twice daily	0.1313 per 1mg
Durvalumab	Durvalumab, 1500mg	Intravenously every 4 weeks	5.2495 per 1mg
Lenvatinib	Lenvatinib, 8mg	Orally once daily	3.9178 per 1mg
Linifanib	Linifanib, 17.5mg	Orally once daily	10.3421 per 1mg
Nivolumab	Nivolumab, 240mg	Intravenously every 2 weeks	3.4926 per 1mg
Sunitinib	Sunitinib, 37.5mg	Orally once daily	0.8329 per 1mg
Tislelizumab	Tislelizumab, 200mg	Intravenously every 3 weeks	2.1040 per 1mg
Sorafenib	Sorafenib, 400mg	Orally twice daily	0.0721 per 1mg
Atezolizumab plus Bevacizumab	Atezolizumab, 1,200 mg	Intravenously every 3 weeks	0.9915 per 1mg
	Bevacizumab, 15 mg/kg		0.0721 per 1mg
Cabozantinib plus Atezolizumab	Cabozantinib, 40mg	Orally once daily	2.3512 per 1 mg

	Atezolizumab, 1,200 mg	Intravenously every 3 weeks	0.9915 per 1mg
Camrelizumab plus Rivoceranib	Camrelizumab, 200mg	Intravenously every 2 weeks	1.9885 per 1 mg
	Rivoceranib, 250mg	Orally once daily	0.0608 per 1mg
Durvalumab plus Tremelimumab	Durvalumab, 1500mg	Intravenously every 4 weeks	1.1665 per 1mg
	Tremelimumab, 300mg		NA[a]
Pembrolizumab plus Lenvatinib	Pembrolizumab, 200mg	Intravenously every 3 weeks (Maximum 2 years)	3.2499 per 1mg
	Lenvatinib, 8mg	Orally once daily	3.9178 per 1mg
Sintilimab plus IBI305	Sintilimab, 200 mg	Intravenously every 3 weeks	1.5671 per 1mg
	IBI305, 15 mg/kg		1.6368 per 1mg
Sorafenib plus Erlotinib	Sorafenib, 400mg	Orally twice daily	0.0721 per 1mg
	Erlotinib, 150mg	Orally once daily	0.0878 per 1mg
Sorafenib plus Doxorubicin	Sorafenib, 400mg	Orally twice daily	0.0721 per 1mg
	Doxorubicin, 60 mg/m^2	Intravenously every 3 weeks	0.3326 per 1mg
Regorafenib	Regorafenib, 160mg	Orally once daily every 4 weeks (d1-8)	0.6257 per 1mg

[a] The drug is unmarketed.

Abbreviation: IBI305, bevacizumab biosimilar.

eTable 5. Summary of statistical goodness-of-fit of K-M curve.

	Exponential	Weibull	Gompertz	Log-logistic	Log-normal
**Sorafenib OS curve**					
AIC	123.3383	117.6602	126.1679	113.4560	115.4887
BIC	126.4490	119.2156	129.2786	116.5667	118.5994
**Sorafenib OS curve**					
AIC	135.2174	115.8270	105.4416	138.2016	108.1548
BIC	136.4363	118.2648	107.8794	140.6394	110.5926

Abbreviation: OS, overall survival; PFS, progression-free survival; AIC, Akaike's information criterion; BIC, Bayesian information criterion.

As for the eight curves listed in the table, the lognormal and log-logistic distribution had the lowest AIC and BIC. While the AIC and BIC tests is important to determine which models fit the observed data best, it does not tell us anything about how suitable a parametric model is for the time period beyond the final trial follow-up. They described the internal validity of fitted models, but not their external validity. The Log-logistic and lognormal models can incorporate non-monotonic hazards but typically have long tails due to a reducing hazard as time increases after a certain point. Actually, the visual fits of the eight curves (eFigure 3) showed that lognormal and log-logistic distribution extended tail, which would likely overestimate OS and PFS in the long term based on clinical experts' opinion.

Weibull distributions are flexible and wildly used in cancer survival analyses. Therefore, the Weibull distributions was likely to be the most reasonable parametric survival model besides the log-logistic and lognormal distribution.

eTable 6. Characteristics of RCTs Included in the Study.

Study	Phase	Trial name	Total sample size	Drug	Sample size	Control	Sample size	Median OS (months)	HR for OS (95% CI)	Median PFS (months)	HR for PFS (95% CI)	Patients with all grade AEs, n (%)	Patients with grade 3 or higher AEs, n (%)
Cheng, 2013	III	A6181170	1,074	Sunitinib	530	Sorafenib	544	7.9 vs 10.2	1.30 (1.13 to 1.50)	3.6 vs 3.0	1.13 (0.99 to 1.30)	NA[a]	432 (82.1) vs 402 (74.2)
Johnson, 2013	III	CA182-033	1,155	Brivanib	577	Sorafenib	578	9.9 vs 9.5	1.07 (0.94 to 1.23)	4.1 vs 4.2	1.01 (0.88 to 1.16)	99 (17.2) vs 98 (17.0)	65 (11.3) vs 67 (11.7)
Zhu, 2014	III	SEARCH	720	Sorafenib plus Erlotinib	358	Sorafenib	362	9.5 vs 8.5	0.93 (0.78 to 1.11)	3.2 vs 4.0	1.11 (0.94 to 1.31)	337 (95.0) vs 345 (95.2)	298 (83.9) vs 315 (87.0)
Cainap, 2015	III	M10-963	1,035	Linifanib	514	Sorafenib	521	9.1 vs 9.8	1.05 (0.90 to 1.22)	5.4 vs 4.0	0.76 (0.64 to 0.90)	483 (94.7) vs 481 (92.7)	435 (85.3) vs 389 (75.0)
Kudo, 2018	III	E7080-G000-304	954	Lenvatinib	478	Sorafenib	476	13.6 vs 12.3	0.92 (0.79 to 1.06)	7.4 vs 3.4	0.66 (0.57 to 0.77)	447 (94.0) vs 452 (95.0)	270 (57.0) vs 231 (49.0)
Abou-Alfa, 2019	III	CALGB 80802	356	Sorafenib plus Doxorubicin	180	Sorafenib	176	9.3 vs 9.4	1.03 (0.82 to 1.29)	4.0 vs 3.7	0.93 (0.75 to 1.16)	NA[a]	NA[a]

Cheng, 2021	III	IMbrave150	501	Atezolizumab plus Bevacizumab	336	Sorafenib	165	19.2 vs 13.4	0.66 (0.52 to 0.85)	6.9 vs 4.3	0.65 (0.53 to 0.81)	284 (86.0) vs 148 (95.0)	143 (43.0) vs 72 (46.0)
Qin, 2021	III	ZGDH3	668	Donafenib	328	Sorafenib	331	12.1 vs 10.3	0.83 (0.70 to 0.99)	3.7 vs 3.6	0.91 (0.76 to 1.08)	314 (94.0) vs 321 (97.0)	125 (38.0) vs 165 (50.0)
Ren, 2021	III	ORIENT-32	571	Sintilimab plus IBI305	381	Sorafenib	191	NR vs 10.4	0.57 (0.43 to 0.75)	4.6 vs 2.8	0.56 (0.46 to 0.70)	337 (89.0) vs 173 (94.0)	128 (34.0) vs 66 (36.0)
Yau, 2021	III	CheckMate 459	743	Nivolumab	371	Sorafenib	372	16.4 vs 14.7	0.85 (0.72 to 1.02)	3.7 vs 3.8	0.93 (0.79 to 1.10)	257 (70.0) vs 338 (93.1)	82 (22.3) vs 180 (49.6)
Abou-Alfa, 2022	III	HIMALAYA	1,171	Durvalumab	389	Sorafenib	389	16.6 vs 13.8	0.86 (0.73 to 1.03)	3.65 vs 4.07	1.02 (0.88 to 1.19)	202 (52.1) vs 317 (84.8)	50 (12.9) vs 138 (36.9)
Abou-Alfa, 2022	III	HIMALAYA	1,171	Durvalumab plus Tremelimumab	393	Sorafenib	389	16.4 vs 13.8	0.78 (0.65 to 0.92)	3.78 vs 4.07	0.90 (0.77 to 1.05)	294 (75.8) vs 317 (84.8)	100 (25.8) vs 138 (36.9)
Finn, 2022	III	LEAP-002	794	Pembrolizumab plus Lenvatinib	395	Lenvatinib	399	21.2 vs 19.0	0.84 (0.71 to 0.99)	8.2 vs 8.1	0.83 (0.71 to 0.98)	381 (96.5) vs 378 (95.7)	247 (62.5) vs 227 (57.5)
Kelley, 2022	III	COSMIC-312	837	Cabozantinib plus Atezolizumab	432	Sorafenib	217	15.4 vs 15.5	0.90 (0.69 to 1.18)	3.7 vs 3.6	0.63 (0.4 to 0.91)	399 (93.0) vs 186 (90.0)	236 (55.0) vs 68 (32.9)

Qin, 2022	III	Rationale-301	674	Tislelizumab	342	Sorafenib	332	15.9 vs 14.1	0.85 (0.71 to 1.02)	2.1 vs 3.4	1.11 (0.92 to 1.33)	259 (76.6) vs 311 (96.0)	75 (22.2) vs 173 (53.4)
Qin, 2022	III	SHR-1210-III-310	543	Camrelizumab plus Rivoceranib	272	Sorafenib	271	22.1 vs 15.2	0.62 (0.49 to 0.80)	5.6 vs 3.7	0.52 (0.41 to 0.65)	265 (97.4) vs 249 (92.6)	220 (80.9) vs 141 (52.4)

[a] Clinical studies are not reported in detail.

Abbreviation: OS, Overall Survival; PFS, progression-freesurvival; CI, confidence interval; AEs, adverse events; IBI305, bevacizumab biosimilar.

eTable 7. Risk of Bias Summary.

Study	Randomization	Allocation concealment	Blinding of participants and personnel	Blinding of outcome assessment	Incomplete outcome data	Selecting report	Other sources of bias
Cheng, 2013	Low	Low	High	High	Low	Low	Low
Johnson, 2013	Low	Low	Low	Low	Low	Low	Low
Zhu, 2014	Low	Low	Low	Unclear	Low	Low	Low
Cainap, 2015	Low	Low	High	Low	Low	Low	Low
Kudo, 2018	Low	Low	High	Low	Low	Low	Low
Abou-Alfa, 2019	Low	Low	High	Unclear	Low	Low	Low
Cheng, 2021	Low	Low	High	Low	Low	Low	Low
Qin, 2021	Low	Low	High	Low	Low	Low	Low
Ren, 2021	Low	Low	High	Low	Low	Low	Low
Yau, 2021	Low	Low	High	Low	Low	Low	Low
Abou-Alfa, 2022	Low	Low	High	Unclear	Low	Low	Unclear
Abou-Alfa, 2022	Low	Low	High	Unclear	Low	Low	Unclear
Finn, 2022	Low	Low	Low	Low	Low	Low	Unclear
Kelley, 2022	Low	Low	High	Low	Low	Low	Low
Qin, 2022	Low	Low	High	Low	Low	Low	Unclear
Qin, 2022	Low	Low	High	Low	Low	Low	Unclear

Low indicates no risk, high indicates high risk and unclear indicates unknown risk.

eTable 8. Pairwise Comparison of ICER ($/QALY).

**Atezolizumab plus Bevacizumab**															
5,838	**Brivanib**														
37,089	Dominated[a]	**Cabozantinib plus Atezolizumab**													
442,000	Dominated	Dominated	**Camrelizumab plus Rivoceranib**												
126,549	Dominated	Dominated	35,554	**Donafenib**											
106,142	Dominated	Dominated	21,330	Dominant[b]	**Durvalumab**										
90,213	Dominated	658,512	28,568	17,756	44,977	**Lenvatinib**									
19,287	Dominated	Dominant	Dominant	Dominant	Dominant	Dominant	**Linifanib**								
114,800	Dominated	Dominated	27,157	Dominant	Dominated	31,237	Dominated	**Nivolumab**							
104,089	Dominated	Dominated	Dominant	231,661	112.073	73,352	Dominated	152,253	**Pembrolizumab plus Lenvatinib**						
Dominated	Dominated	Dominant	56,769	46,573	38,559	39,745	Dominated	41,989	23,917	**Sintilimab plus IBI305**					
76,955	Dominated	228,512	22,848	9,419	25,005	Dominant	Dominated	17,509	52,410	34,959	**Sorafenib**				
51,762	Dominated	81,893	3,703	Dominant	Dominant	Dominant	Dominated	Dominant	12,572	16,370	Dominant	**Sorafenib plus Doxorubicin**			
65,742	Dominated	182,381	9,076	Dominant	Dominant	Dominant	Dominated	Dominant	29,553	30,115	880,125	Dominated	**Sorafenib Plus Erlotinib**		
50,308	Dominated	63,385	15,747	7,019	12,749	2,744	Dominated	10,142	24,032	27,441	5,302	46,183	25,185	**Sunitinib**	
129,222	Dominated	Dominated	47,199	163,654	Dominated	Dominated	Dominated	982,500	210,859	51,942	Dominated	Dominated	Dominated	Dominated	**Tislelizumab**

[a] One strategy showed lower effectiveness and high cost, as compared with another strategy (Dominated). [b] One strategy showed higher effectiveness and lower cost, as compared with another strategy (Dominant).
